# Development and validation of a digital learning resource quality evaluation framework: a mixed-methods study in health professions education

**DOI:** 10.3389/fpubh.2026.1958724

**Published:** 2026-10-14

**Authors:** Wenlong Cui, Yuan Zhang, Wenlin Dong, Yang Xu, Fang Xu, Tiantian Zhao, Yan Zhang, Lingli Dong, Hua Bai, Dingyun You, Huixin Wu

**Affiliations:** 1 School of Public Health, Kunming Medical University, Kunming, China; 2 Academy of Biomedical Engineering, Kunming Medical University, Kunming, China

**Keywords:** analytic hierarchy process, Delphi method, digital learning resources, health professions education, quality evaluation

## Abstract

**Background:**

Digital learning resources (DLRs) are now core teaching tools in health professions education, yet quality evaluation remains constrained by subjective weighting bias and a persistent gap between educational importance and operational measurability. Because DLRs increasingly mediate what and how students learn, deficiencies in resource quality propagate directly into competency attainment, making quality evaluation a proximal determinant of student competency outcomes. This study aimed to develop and validate a practical DLR quality evaluation framework for medical and public health education.

**Methods:**

A sequential mixed-methods design was employed. First, a modified Delphi procedure with 26 experts was used to construct the hierarchical indicator system. Second, an expert-based and data-derived integrated weighting model combining AHP and EWM was developed to balance expert judgment and data discriminatory power. Third, a measurability correction mechanism and a four-quadrant prioritization matrix were established. The framework was validated with 10 DLRs across 10 medical courses, involving 309 students.

**Results:**

The final system comprised 4 primary dimensions, 9 secondary indicators, and 14 tertiary indicators. Content Quality had the highest integrated weight (0.469), with Data Visualization (0.136) and Updated Content and Frontier Knowledge (0.103) as the top tertiary indicators. The four-quadrant matrix classified 7 indicators for direct implementation and 2 for targeted methodological development. Validation confirmed high internal consistency (Cronbach’s *α* = 0.987) and satisfactory content validity (S-CVI = 0.951); absolute-agreement inter-rater ICCs were near zero, indicating substantial between-student variability in scoring levels.

**Conclusion:**

This study provides a reliable, implementable DLR quality evaluation framework. By converting evaluation results into ranked, measurable improvement priorities, the framework directs resource revision toward the attributes that bear most directly on student competency development. The methodological workflow shows potential for transferability across nursing, pharmacy, public health and other health-related disciplines via contextual calibration, pending formal cross-context validation.

## Introduction

The global digital transformation of higher education has fueled the rapid proliferation of digital learning resources (DLRs), including massive open online courses, virtual simulations, digital case libraries, and interactive learning modules (1, 2). While these resources have substantially reduced temporal and spatial barriers to learning and expanded access to educational opportunities worldwide, the sector faces a pervasive, unresolved challenge: the transition from large-scale resource construction to systematic, evidence-based quality management (1, 3). As DLRs become increasingly central to formal teaching and learning, inconsistent resource quality has emerged as a critical risk to learning outcomes across all higher education disciplines. Establishing rigorous, implementable quality evaluation mechanisms has therefore become a shared priority for global higher education governance (4).

Numerous evaluation frameworks and rubrics have been developed for digital learning resources in general higher education, among which the Rubric for Open Educational Resources (ROER) and the technology-enhanced learning (TEL) evaluation framework are widely cited representatives (5, 6). These frameworks have laid foundational structural dimensions for DLR quality assessment and advanced the standardization of evaluation practice. Nevertheless, three persistent methodological gaps remain in mainstream DLR evaluation research, limiting the real-world utility and disciplinary adaptability of existing tools.

First, prevailing generic evaluation frameworks are designed for generalized higher education scenarios and lack formal discipline-specific calibration mechanisms (3). While one-size-fits-all rubrics offer broad applicability, they struggle to balance universal pedagogical principles with the rigorous professional standards required in vocation-oriented disciplines. For high-stakes professional fields where content accuracy directly impacts practice safety, generic frameworks often fail to capture domain-specific quality requirements such as alignment with industry standards and professional competency outcomes, resulting in insufficient evaluation discriminative power (6, 7).

Second, indicator weighting in most existing frameworks relies predominantly on subjective expert judgment, which is prone to a pronounced ceiling effect: experts tend to rate most pedagogically relevant indicators as similarly important, resulting in poor differentiation of core quality drivers and diluted priority setting (8). The methodological integration of subjective expert authority and objective data discriminatory power remains underdeveloped, and few studies have systematically reconciled the tension between value-based priority setting and data-driven weight differentiation (9–11).

Third, there is a widespread disconnection between the theoretical importance of evaluation indicators and their operational measurability. Educationally critical indicators (e.g., student competency development, higher-order thinking cultivation) are often abstract and difficult to quantify objectively, whereas easily measurable technical metrics carry limited educational value (12). Few evaluation frameworks incorporate a standardized measurability correction mechanism or provide stratified implementation strategies based on dual dimensions of importance and feasibility, leaving many theoretically sound rubrics difficult to deploy in routine quality management practice.

These gaps are widening under generative artificial intelligence. AI-assisted authoring allows digital learning materials to be produced and revised at a pace that conventional expert review cannot match, while the resulting content carries a non-trivial error burden: a systematic review of AI-generated medical education videos found that every included study reported content inaccuracies or bias, with the comparative accuracy assessment showing control materials outperforming generated materials on script and image accuracy (13). Detection is also unreliable: in a multicentre study, general practice trainees identified only 55.0% of ChatGPT-4o hallucinations, and model accuracy fell to 44.2% on professional-practice items after human verification (14). Content validity therefore ceases to be a property established once at resource creation and becomes a condition requiring continuous, structured verification, reinforcing the need for explicit accuracy and currency criteria within an evaluation framework rather than reliance on expert judgment alone.

As a typical high-stakes, standards-intensive professional discipline, medical education presents an ideal test bed for validating optimized DLR evaluation methodologies. Medical education demands strict adherence to scientific accuracy, clinical practice alignment, and professional ethical standards, and its quality requirements are far more stringent than those of many general education disciplines (15, 16). If an evaluation framework can demonstrate robust reliability and validity in the demanding medical education context, its methodological rigor and transferability to other higher education disciplines will be strongly supported. Against this backdrop, medical education serves not as the sole boundary of this research, but as a demanding empirical context for examining the methodological robustness of the proposed evaluation approach, with implications for its potential transferability to other disciplines.

The choice of AHP and EWM reflects the task at hand: weighting a fixed indicator hierarchy rather than ranking discrete alternatives. AHP decomposes expert judgment along the same hierarchy used for indicator construction and provides an established consistency diagnostic (CR < 0.10) for identifying unreliable respondents; EWM derives weights from the dispersion of the rating matrix and thus counteracts the ceiling effect inherent in purely judgment-based methods. Outranking and distance-based techniques such as TOPSIS, VIKOR and ELECTRE are designed to rank alternatives rather than to weight indicators, whereas the Best–Worst Method, DEMATEL and ANP require denser pairwise input than a 26-member panel can reliably supply.

Measurability correction refers to the recalibration of each indicator’s integrated weight by its measurability score, where measurability denotes the extent to which an indicator can be observed, quantified, or evidenced through objective platform data rather than subjective judgment. This correction yields a final actionable weight that reflects both how much an indicator matters educationally and how readily it can be assessed in practice.

To address these gaps, this study aims to develop and validate a practical quality evaluation framework for digital learning resources in health professions education, integrating educational importance with operational feasibility. A sequential mixed-methods design is employed, featuring two core innovations: (1) an expert-based and data-derived integrated weighting model combining AHP and EWM to mitigate ceiling effects in expert judgment; and (2) a formal measurability correction mechanism paired with a four-quadrant prioritization matrix to guide phased implementation. Empirically tested in medical and preventive medicine education, the framework provides a replicable quality governance tool for health professions education and can be calibrated for other health-related disciplines.

## Methods

The methodological workflow proposed in this study is generally applicable to DLR quality evaluation across higher education; this paper conducts indicator construction, weight calibration and systematic validation using medical higher education as the empirical scenario. A sequential mixed-methods design was employed, integrating a modified Delphi consensus process, a dual expert-based and data-derived weighting model, and a measurability correction mechanism to develop and validate the evaluation framework. The study was conducted in accordance with the Declaration of Helsinki, with ethical approval granted by the Medical Ethics Committee of Kunming Medical University (Approval Number: KMU2026MEC001). All participants provided implied informed consent via voluntary completion and return of anonymous questionnaires. The overall methodological framework is summarized in Figure 1.

**Figure 1:**
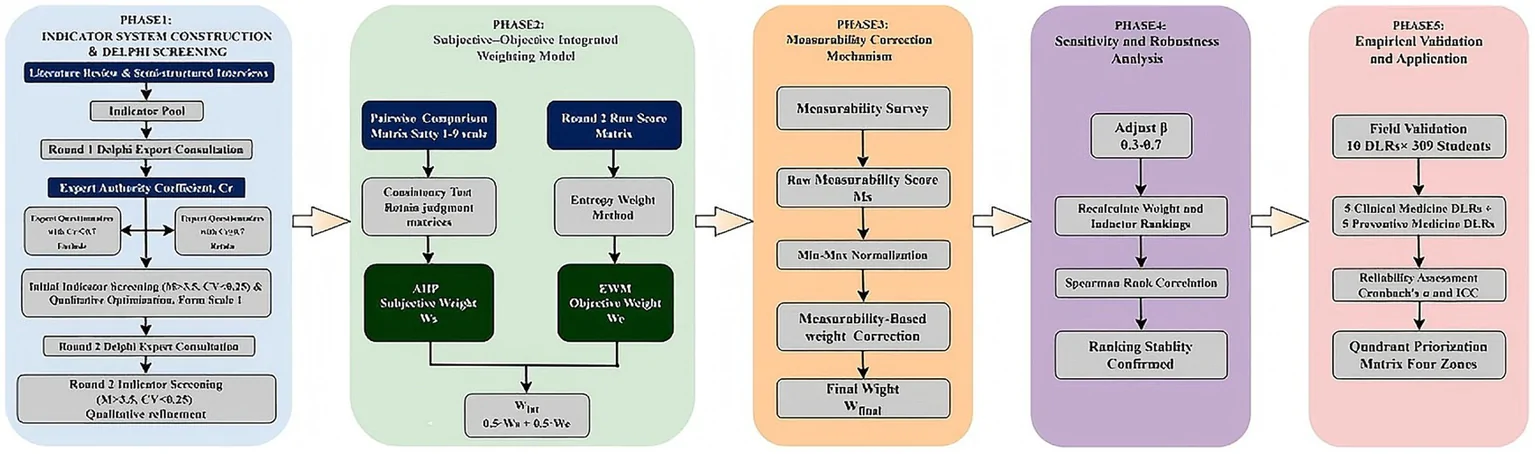
Methodological framework of the study. The methodological framework comprises five components: (1) modified Delphi consensus to establish the indicator system, (2) AHP-EWM dual weighting integrating expert-based and data-derived weights, (3) measurability correction to produce final implementation weights, (4) sensitivity analysis to verify weight stability, and (5) field validation with 10 digital learning resources and 309 students.

### Modified Delphi process for Indicator system construction

This phase aimed to establish the structural validity of the evaluation system through initial pool construction and two rounds of anonymous expert consultation.

#### Initial indicator pool construction

An initial indicator pool was developed via a two-step mixed approach: (1) a systematic literature search in Web of Science Core Collection, OpenAlex, PubMed, and CNKI (January 2019 to December 2024), combining three concept blocks via Boolean operators: (“digital learning resource” OR “digital teaching resource” OR “online learning resource” OR “open educational resource”) AND (“medical education” OR “health professions education” OR “clinical education”) AND (“quality evaluation” OR “quality assessment” OR “evaluation framework” OR “evaluation indicator”). Eligible studies proposed or applied a structured evaluation framework with explicit indicator dimensions and were published in English or Chinese in peer-reviewed journals. Following duplicate removal, two researchers independently screened titles/abstracts and full texts, with disagreements resolved by a third senior researcher. Screening of 1,162 initial records (1,090 after deduplication) yielded 273 full-text articles, of which 248 studies were included for indicator extraction (PRISMA flow diagram, Supplementary material 1). Candidate indicators were extracted independently by two researchers and synthesized into a preliminary dimensional structure; (2) semi-structured focus group interviews with 15 stakeholders (medical education researchers, clinical experts, frontline teachers, and education administrators, all with at least 5 years of relevant experience) and 50 medical students, to supplement medicine-specific indicators (Semi-structured focus group interview guides in Supplementary material 2). The initial pool comprised 5 primary, 11 secondary, and 21 tertiary indicators. Focus group interviews followed a semi-structured guide developed by the research team based on the literature-derived dimensions and pilot-tested with two medical education experts. Stakeholder interviews comprised 4 focus groups (stratified by role), each lasting 45–60 min; student interviews comprised 5 focus groups (10 students per group), each lasting 30–45 min. All sessions were audio-recorded with consent and transcribed verbatim. Data saturation was judged when no new indicator categories emerged across consecutive groups.

Transcripts were reviewed independently by two researchers to identify and extract candidate indicators, using the literature-derived indicator pool as an initial coding frame. Any discrepancies in indicator identification or categorization were resolved through discussion within the research team until consensus was reached. The extracted indicators were merged with the literature-derived pool to form the initial indicator pool for Delphi consultation (Table 1).

**Table 1:** Demographic characteristics of focus group interview participants.

Category	Role/major	Number	Experience (years)
Experts	Medical education researchers	4	18.5 ± 6.2
	Clinical medicine experts	4	15.3 ± 5.8
	Frontline teaching experts	4	12.7 ± 4.3
	Education administrators	3	14.0 ± 5.1
Students	Clinical medicine	23	N/A
	Preventive medicine	27	N/A
Total		65	

All stakeholder participants had at least 5 years of relevant experience. The 50 medical students were enrolled in Years 1 through 4 at Kunming Medical University, spanning clinical medicine (46%) and preventive medicine (54%) programs (Detailed grade distribution of the 50 student participants is provided in Supplementary material 3).

#### Expert panel selection

A maximum variation purposive sampling strategy was used to recruit a multi-stakeholder expert panel, with inclusion criteria: (1) intermediate or higher professional title; (2) at least 5 years of experience in medical education, clinical practice, or education administration; (3) demonstrated familiarity with digital medical education resource construction. The final panel covered four domains: medical education researchers, educational administrators, clinical medical experts, and frontline teaching experts, to ensure multi-dimensional representativeness.

#### Delphi consultation implementation and screening criteria

A two-round anonymous Delphi consultation was conducted, with strict anonymity maintained throughout: all experts were blinded to the identity and responses of other panel members, with only one-way communication between the research team and individual experts.

The Round 1 questionnaire consisted of three sections (see Supplementary material 4): (1) expert demographic information; (2) self-assessment scales for judgment basis (Ca) and familiarity with the topic (Cs); (3) a 5-point Likert scale (1 = completely unnecessary to 5 = highly necessary) for experts to rate the importance of each indicator, with space for qualitative modification suggestions.

After retrieving Round 1 questionnaires, the Expert Authority Coefficient (Cr) was calculated as Cr = (Ca + Cs) /2. Responses from any expert with Cr < 0.70 were excluded. Indicators were retained only if they met dual thresholds: a mean importance score (*M*) > 3.50 and a coefficient of variation (CV) < 0.25 on a 5-point scale. These thresholds follow conventional Delphi practice in health professions education (12, 17): M > 3.50 corresponds to a mean rating above “moderately necessary,” and CV < 0.25 indicates acceptable dispersion.

Combining these quantitative results with qualitative expert feedback, the initial framework was revised. After sharing aggregated first-round results with experts as feedback, the Round 2 questionnaire was distributed (see Supplementary material 4).

Upon retrieval of Round 2 questionnaires, the same screening criteria (*M* > 3.50 and CV < 0.25) were applied. Through this iterative convergence process, the optimized hierarchical framework was finalized.

### Expert-based and data-derived integrated weighting model

Utilizing the finalized indicator structure and expert consensus data from Round 2, an Integrated Weight (*W*_int_) was established by combining AHP with EWM.

#### Data consistency filtering

Prior to weight calculation, a consistency check was performed on expert pairwise comparison matrices (constructed using Saaty’s 1–9 scale) for AHP (18). Only matrices with a Consistency Ratio (CR) < 0.10 were retained for final weight calculation.

#### Subjective weighting (AHP)

The subjective weight vector was derived from the normalized principal eigenvector of validated consistent judgment matrices, reflecting expert-assigned pedagogical priorities in medical education (AHP Calculation Step see Supplementary material 5).

#### Data-derived weighting (EWM)

To address the ceiling effect where experts may rate all medical indicators as highly important, EWM was applied to the Round 2 raw score matrix to calculate data-derived weights based on score dispersion; indicators with higher variance have greater discriminatory power (11) (EWM Calculation Step see Supplementary material 6).


$$\mathrm{ej}=-k\sum_{i=1}^{n}p_{\mathrm{ij}}\ln(p_{\mathrm{ij}}),\text{where}\;k=\frac{1}{\ln n}$$

$$W_{\mathrm{EWM}}=\frac{1-e_{j}}{\sum_{j=1}^{m}1-e_{j}}\;$$

#### Integrated weight fusion

The final integrated weight (*W*_int_) for each indicator was calculated as the arithmetic mean of AHP and EWM weights, with an equal weighting coefficient (*β* = 0.5), following the combined weighting approach applied in prior medical evaluation research (19). Sensitivity analysis was conducted by varying *β* from 0.3 to 0.7 to assess the stability of indicator rankings.


$$W_{\mathrm{int}}=\beta \times W_{\mathrm{AHP}}+(1-\beta )\times W_{\mathrm{EWM}}$$

### Measurability correction mechanism

To address the gap between theoretical importance and practical implementation, a measurability correction mechanism was introduced. Experts rated the measurability of each tertiary indicator on a 5-point scale, defined as the extent to which an indicator could be observed, quantified, or evidenced through objective platform data rather than subjective judgment (20). Raw measurability scores (range 1–5) were normalized to the [0,1] interval using the Min-Max method.


$$Ms_{\text{normalized}}=\frac{Ms_{\mathrm{raw}}-\min(\mathrm{Ms})}{\max(\mathrm{Ms})-\min(\mathrm{Ms})}$$

### The four-quadrant prioritization matrix

Indicators were mapped to a coordinate system with integrated weight (*W*_int_, *Y*-axis) and raw measurability score (*Ms*, *X*-axis), using pre-specified thresholds (weight = 0.06, measurability raw score = 4.0) to classify indicators into four zones (12, 21). Both thresholds were determined *a priori* by the research team in consultation with 3 senior medical education experts prior to data analysis. The measurability threshold of 4.0 was defined as the minimum score at which an indicator could be predominantly assessed via objective platform data rather than subjective expert judgment, and the weight threshold of 0.06 was set as a pragmatic cut-off for distinguishing core indicators from supporting ones. The stability of indicator classification near these thresholds was examined by varying the cut-offs within pre-specified ranges (measurability: 3.8–4.2). The four zones were defined as follows: Quadrant I (High Importance, High Measurability) for indicators suitable for direct implementation; Quadrant II (High Importance, Low Measurability) for indicators requiring targeted methodological development; Quadrant III (Low Importance, Low Measurability) for indicators requiring optimization; and Quadrant IV (Low Importance, High Measurability) for supplementary indicators.

### Final actionable weight calculation

The final weight (*W*_final_) for the scoring instrument was calibrated by adjusting the integrated weight with the normalized measurability score, using an adjustment coefficient (*γ* = 0.5) to maintain the primacy of educational value while rewarding measurability, consistent with combined correction models in prior evaluation research (22). The impact of *γ* on final rankings was examined via sensitivity analysis across *γ* = 0.3, 0.5, and 0.7.


$$W_{\text{final}}=W_{\mathrm{int}}\times [1+\gamma \times (Ms_{\text{normalized}}-0.5)]$$

### Sensitivity analysis

Sensitivity analysis was conducted by adjusting the proportional weight of subjective AHP weighting (*β*) from 0.3 to 0.7. Spearman’s rank correlation analysis was used to assess the consistency of indicator rankings across different *β* settings.

### Validation of the evaluation system

The final evaluation system was validated in two steps: content validity was assessed by the Delphi expert panel, and reliability was assessed in a student survey. The student survey covered ten digital learning resources (DLRs), one from each of five clinical-medicine courses (Diagnostic imaging, Diagnostics, Clinical skills, Surgery, and Internal medicine) and five preventive-medicine courses (Sanitary chemistry, Sanitary microbiology, Medical statistics, Epidemiology, and Toxicology) at Kunming Medical University, spanning basic-to-core curricula and multiple resource formats, including virtual simulations, digital case libraries, and MOOCs (the scale is provided in Supplementary material 4).

#### Participants

Two groups participated in the validation. Content validity was assessed by the 26 experts of the Delphi panel. Reliability was assessed by undergraduate medical students recruited from the clinical-medicine and preventive-medicine programs who had used the selected DLRs for at least 8 weeks. Each student evaluated the DLR used in their own course; each of the 10 courses had one designated DLR. The final sample comprised 309 students (156 clinical medicine, 153 preventive medicine), with 13–43 students per course.

#### Validity testing

Content validity was assessed using the item-level (I-CVI) and scale-level (S-CVI) content validity indices, based on the Round 2 ratings of the 26 Delphi panel experts. Because the Delphi design did not include a separate relevance-rating step, the I-CVI was operationalized as the proportion of experts rating each indicator’s importance as ≥4 on the 5-point Likert scale (importance-based CVI); the S-CVI was the mean of all item-level values. An I-CVI ≥ 0.78 was regarded as acceptable (23).

#### Reliability testing

Reliability of the 14 tertiary indicators was assessed in the student sample (*N* = 309) using intraclass correlation coefficients (ICCs) and Cronbach’s alpha. For each course, ICC(2,1)—a two-way random-effects, single-rater, absolute-agreement ICC (24)—was computed to assess inter-rater consistency among students evaluating the same DLR, with students treated as raters and DLRs (across 14 indicators) as targets; 95% confidence intervals were derived from the corresponding F statistics (25). Pooled ICC(2,1) values were computed across (a) the five clinical-medicine courses, (b) the five preventive-medicine courses, and (c) all ten courses, using a mixed-effects model with course as a random effect that appropriately weighted each course by its sample size (rather than simple arithmetic averaging). Internal consistency was assessed with Cronbach’s alpha (26).

## Results

### Expert panel characteristics and structural validity

A total of 28 experts were invited for Round 1 Delphi consultation; 26 valid responses were included after excluding one non-responder and one expert with authority coefficient (Cr) < 0.70. The sample size is consistent with Delphi methodological recommendations that 15 to 30 experts are sufficient to achieve stable consensus (12, 17). Fifteen of 26 experts (57.7%) passed the AHP consistency check (CR < 0.10); sensitivity analyses showed no material impact of consistency filtering on the resulting weights (Supplementary material 7).

Of the 26 panelists, 15 (57.7%) were professor-level senior professionals, with a mean age of 51.0 ± 11.8 years and mean working experience of 27.20 ± 12.19 years. Experts were distributed across four domains: medical education research (*n* = 7), educational administration (*n* = 5), clinical medicine (*n* = 7), and frontline teaching (*n* = 7). The mean expert authority coefficient was 0.87, indicating high credibility (Table 2).

**Table 2:** Distribution of expert demographic characteristics (*n* = 26).

Characteristic	Number	Percentage (%)
Professional field		
Medical education research	7	26.9
Educational administration	5	19.2
Clinical medicine expert	7	26.9
Teaching expert	7	26.9
Professional title		
Senior (professor-level)	15	57.7
Associate senior	9	34.6
Intermediate	2	7.7
Years of work experience		
5–20 years	18	69.2
>20 years	8	30.8

Demographic characteristics of the 26 experts enrolled in the modified Delphi consultation.

The initial indicator pool comprised 5 primary dimensions, 11 secondary indicators, and 21 tertiary indicators. In Round 1, four tertiary indicators were excluded for failing to reach consensus (CV ≥ 0.25)—Accessibility, Interface Aesthetics, Collaborative Learning Function, and Timeliness of Teacher Response (*M* = 3.77–4.04, CV = 0.25–0.26). The remaining 17 tertiary indicators were further reduced to 14 through mergers informed by qualitative expert feedback on conceptual overlap and scenario misalignment; concurrently, the “Artistry and Practicality” primary dimension was dissolved and its indicators redistributed. The revised framework for Round 2 thus comprised 4 primary dimensions, 10 secondary indicators, and 14 tertiary indicators.

Round 2 achieved a 100% response rate, and all indicators met the retention criteria (*M* > 3.50, CV < 0.25). In the post-Round 2 qualitative synthesis, the panel removed the secondary indicator “Collaborative Function” (under Platform Functionality) because it assessed platform-level capability rather than an intrinsic quality attribute of the digital learning resource itself; this reduced the secondary indicators from 10 to 9, while the 14 tertiary indicators remained unchanged. The final framework therefore comprised 4 primary dimensions, 9 secondary indicators, and 14 tertiary indicators with operational definitions (Table 3; item-level statistics for both rounds are reported in Supplementary material 8).

**Table 3:** The optimized evaluation indicator system for medical digital learning resources.

Primary dimension/secondary indicator	Tertiary indicator	Connotation (operational definition)
1. Content Quality	1.1.1 Advancedness	Integration of new medical science concepts and digital pedagogies.
1.1 Educational Philosophy		
	1.1.2 Student-Centeredness	Alignment with student learning outcomes and competency development.
1.2 Content Scientificity	1.2.1 Scientific Accuracy	Comprehensive and error-free presentation of core knowledge, adhering to medical curriculum standards.
	1.2.2 Updated Content and Frontier Knowledge	Incorporation of recent (past 3 years) medical advancements and research findings.
1.3 Professional Ethics and Values	1.3.1 Professional Ethics and Values Education (Ideological and Political Guidance)	Integration of professional ethics, scientific spirit, and humanistic care within medical contexts.
	1.3.2 Social Responsiveness	Focus on primary healthcare needs and public health issues.
2. Instructional Design	2.1.1 Methodological Appropriateness	Compatibility with active learning strategies (e.g., PBL, CBL, TBL).
2.1 Teaching Methods		
2.2 Resource Diversity	2.2.1 Resource Formats	Variety of digital formats (e.g., VR, 3D anatomy, virtual simulations).
2.3 Learning Support	2.3.1 Personalized Learning	Tiered difficulty levels and customizable assessment modules.
	2.3.2 Interaction Design	Interactive elements (discussions, collaborative tasks).
3. Evaluation and Feedback	3.1.1 Multiple Evaluations	Combination of formative and summative assessments.
3.1 Learning Evaluation		
3.2 Application Feedback	3.2.1 Timeliness and Update Adaptability	Inclusion of latest clinical guidelines; modular architecture for updates.
4. Platform Functionality	4.1.1 Compatibility and Security	Cross-platform compatibility; data privacy and copyright compliance.
4.1 Interactive Experience		
	4.1.2 Data Visualization	Real-time tracking and visual feedback of learning progress.

Operational definitions are calibrated to the medical education context for empirical validation. The four primary dimensions are designed to be shared across health professions disciplines, with tertiary indicators adjustable via discipline-specific calibration.

### Weight distribution

Before computing the integrated weights, a consistency filtering was applied to the judgment matrices of the 26 experts from Round 2. Results indicated that 15 experts successfully passed the Consistency Ratio threshold (CR < 0.10), and their judgment matrices were used for weight calculation. To assess whether restricting AHP analysis to the 15 consistent experts introduced selection bias, a sensitivity analysis was conducted comparing (a) the baseline 15-expert AHP weights, (b) weights derived from all 26 experts using geometric-mean aggregation of individual judgment matrices (including inconsistent ones), and (c) bootstrapped weights with 1,000 resamples from the full expert pool. The top two primary dimensions (content quality and instructional design) retained their rank order across all three approaches; the rank order of the two lower-weighted dimensions (evaluation feedback and platform function) reversed after excluding inconsistent experts. The maximum relative weight deviation at the primary-dimension level was 35.00% (evaluation feedback). No systematic differences were found between retained and excluded experts by professional domain (*χ*^2^ = 7.271, *p* = 0.064) or seniority (Mann–Whitney *U* test, *p* = 0.111), suggesting that consistency filtering did not introduce meaningful selection bias (Table 4; Supplementary material 7). The two groups were also comparable in professional field (*p* = 0.064), professional title (*p* = 0.061) and years of professional experience (27.20 ± 12.19 vs. 19.18 ± 10.92 years, *p* = 0.111) (Supplementary material 14; Table S14.1).

**Table 4:** Distribution of weights and measurability scores across indicators.

Hierarchy level	Indicator name	AHP weight	Data-derived weighting (EWM)	Integrated weight	Measurability (raw)	Final weight
Primary	1. Content Quality	0.492	0.445	0.469	4.965	0.475
	2. Instructional Design	0.316	0.184	0.250	4.231	0.248
	3. Evaluation and Feedback	0.078	0.072	0.075	4.081	0.074
	4. Platform Functionality	0.113	0.299	0.206	4.077	0.203
Tertiary	1.1.1 Advancedness	0.078	0.039	0.059	4.427	0.059
	1.1.2 Student-Centeredness	0.136	0.053	0.095	4.886	0.095
	1.2.1 Scientific Accuracy	0.066	0.118	0.092	4.631	0.092
	1.2.2 Updated Content and Frontier Knowledge	0.031	0.176	0.103	4.232	0.103
	1.3.1 Professional Ethics and Values Education (Ideological and Political Guidance)	0.116	0.020	0.068	4.267	0.068
	1.3.2 Social Responsiveness	0.065	0.038	0.051	4.066	0.051
	2.1.1 Methodological Appropriateness	0.145	0.016	0.081	4.982	0.081
	2.2.1 Resource Formats	0.051	0.068	0.060	4.090	0.059
	2.3.1 Personalized Learning	0.037	0.027	0.032	4.011	0.032
	2.3.2 Interaction Design	0.083	0.073	0.078	4.313	0.078
	3.1.1 Multiple Evaluations	0.045	0.041	0.043	4.492	0.043
	3.2.1 Timeliness and Update Adaptability	0.033	0.031	0.032	4.086	0.032
	4.1.1 Compatibility and Security	0.040	0.101	0.071	3.988	0.070
	4.1.2 Data Visualization	0.073	0.198	0.136	3.910	0.135

AHP, analytic hierarchy process; EWM, entropy weight method. Primary-level AHP weights are global weights summing to 1.000. Tertiary-level AHP weights are global weights across all 14 indicators (summing to 1.000), not relative weights within each parent node. Integrated weight is the global comprehensive weight. Measurability scores are raw mean scores (range 1–5); for final weight calculation, these were normalized to [0,1]. Final weights were adjusted using the measurability correction mechanism.

#### Primary dimensions analysis

The Content Quality dimension received the highest integrated weight (Wint = 0.469), followed by Instructional Design (0.250), Platform Functionality (0.206), and Evaluation and Feedback (0.075). While AHP assigned substantially higher weight to Content Quality (0.492) than to Platform Functionality (0.113), EWM assigned the highest weight to Platform Functionality (0.299) and the lowest to Evaluation and Feedback (0.072), indicating that expert ratings showed greater dispersion for platform-related indicators. This difference indicates that experts subjectively value content quality most, while the data-derived (EWM) weights suggest that platform features carry substantial discriminatory power in distinguishing resource quality.

Descriptive statistics of the Round 2 expert rating matrix used for EWM computation (overall *M* = 4.59, SD = 0.59, 64.3% of ratings at the maximum score of 5) are reported in Supplementary material 8 to allow readers to assess the degree of score dispersion underlying the EWM weights.

#### Visualization of weight distribution

To visually trace how the integrated weighting model allocates importance from the four primary dimensions down to specific tertiary indicators, we constructed a Sankey diagram (Figure 2). This visualization serves two purposes: (1) it shows whether weight is concentrated in a single dimension or distributed across multiple dimensions, and (2) it identifies which specific indicators within each dimension dominate the evaluation profile. The diagram confirms that Content Quality subsumes the largest share of total weight (46.9%), and that within this dimension, Student-Centeredness (0.095) and Scientific Accuracy (0.092) are the primary contributors. Across all dimensions, Data Visualization (0.136) and Updated Content and Frontier Knowledge (0.103) carry the widest bands. This hierarchical weight profile directly informs the subsequent four-quadrant classification by establishing which indicators meet the importance threshold.

**Figure 2:**
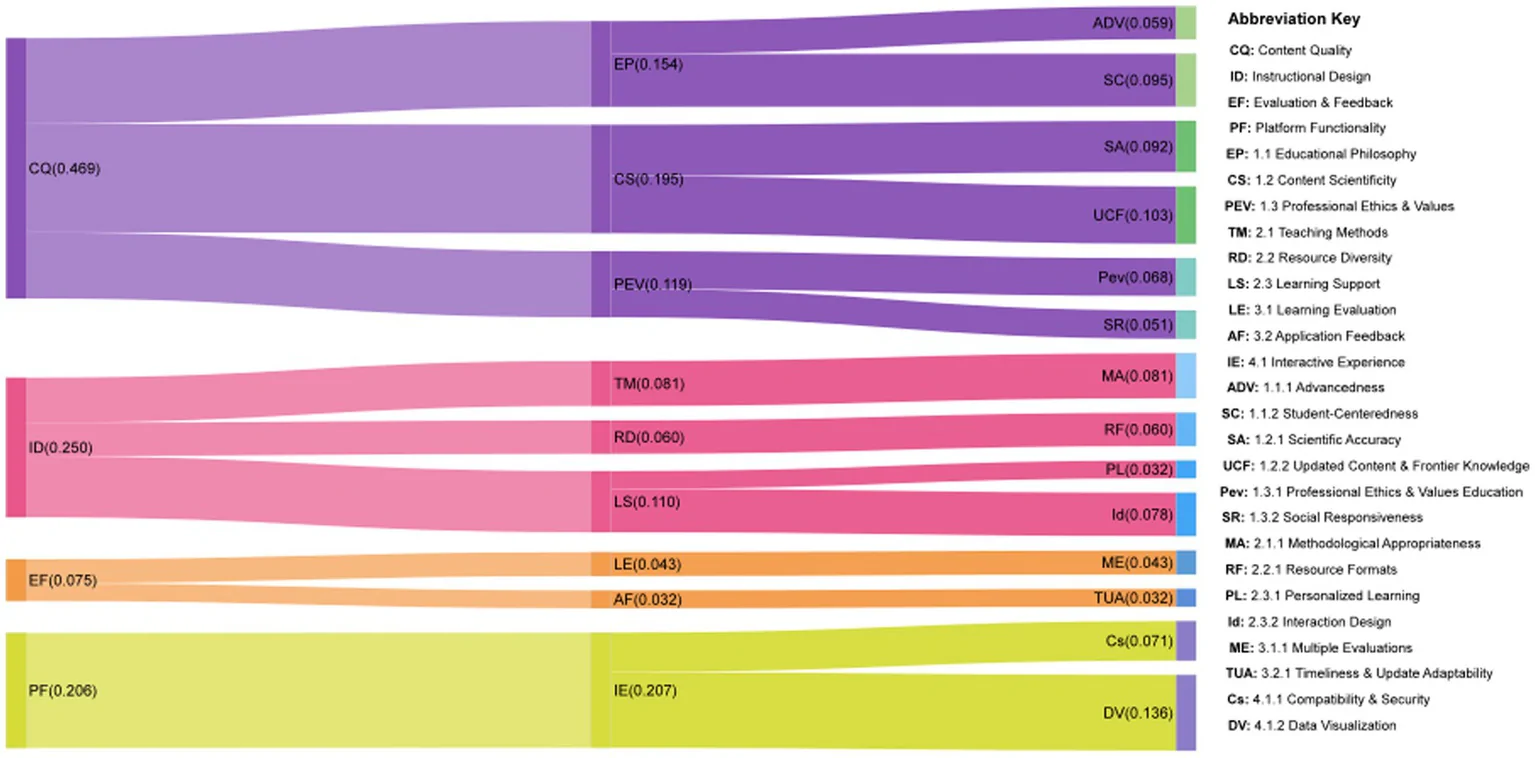
Sankey diagram of integrated weight flow. Band width is proportional to the global integrated weight.

#### Sensitivity analysis results

To assess whether the baseline *β* = 0.5 setting biases evaluation results, we conducted sensitivity analysis using *β* = 0.3 (EWM-dominant) and *β* = 0.7 (AHP-dominant). Figure 3 overlays tertiary indicator weights under the three *β* scenarios as radar polygons, enabling visual inspection of weight stability. Quantitatively, primary-dimension rankings were identical to the baseline at *β* = 0.7 (Spearman’s *ρ* = 1.000, *n* = 4, exact *p* = 0.083); at *β* = 0.3, a single adjacent rank exchange occurred between Instructional Design and Platform Functionality (*ρ* = 0.800, exact *p* = 0.333). We note that the perfect *ρ* = 1.000 at the primary level should be interpreted with caution, as it is based on only four dimensions and statistical significance is less meaningful at such small sample sizes (the minimum achievable exact two-tailed *p*-value at *n* = 4 is 0.083). At the tertiary-indicator level, rank-order correlations with the baseline remained high (*β* = 0.3: *ρ* = 0.928, 95% CI 0.783–0.977, *p* = 1.72 × 10^−6^; *β* = 0.7: *ρ* = 0.858, 95% CI 0.601–0.954, *p* = 8.65 × 10^−5^); complete results for all five *β* settings are given in Supplementary material 14; Table S14.4. Rank changes at non-baseline settings involved 9–12 indicators, predominantly one- to two-position shifts among indicators whose weights differed by less than 0.01; however, five indicators (Advancedness, Methodological Appropriateness, Professional Ethics and Values Education, Resource Formats, and Compatibility and Security) crossed the 0.06 weight threshold at one or more *β* settings and should be interpreted as specification-sensitive (Supplementary material 9).

**Figure 3:**
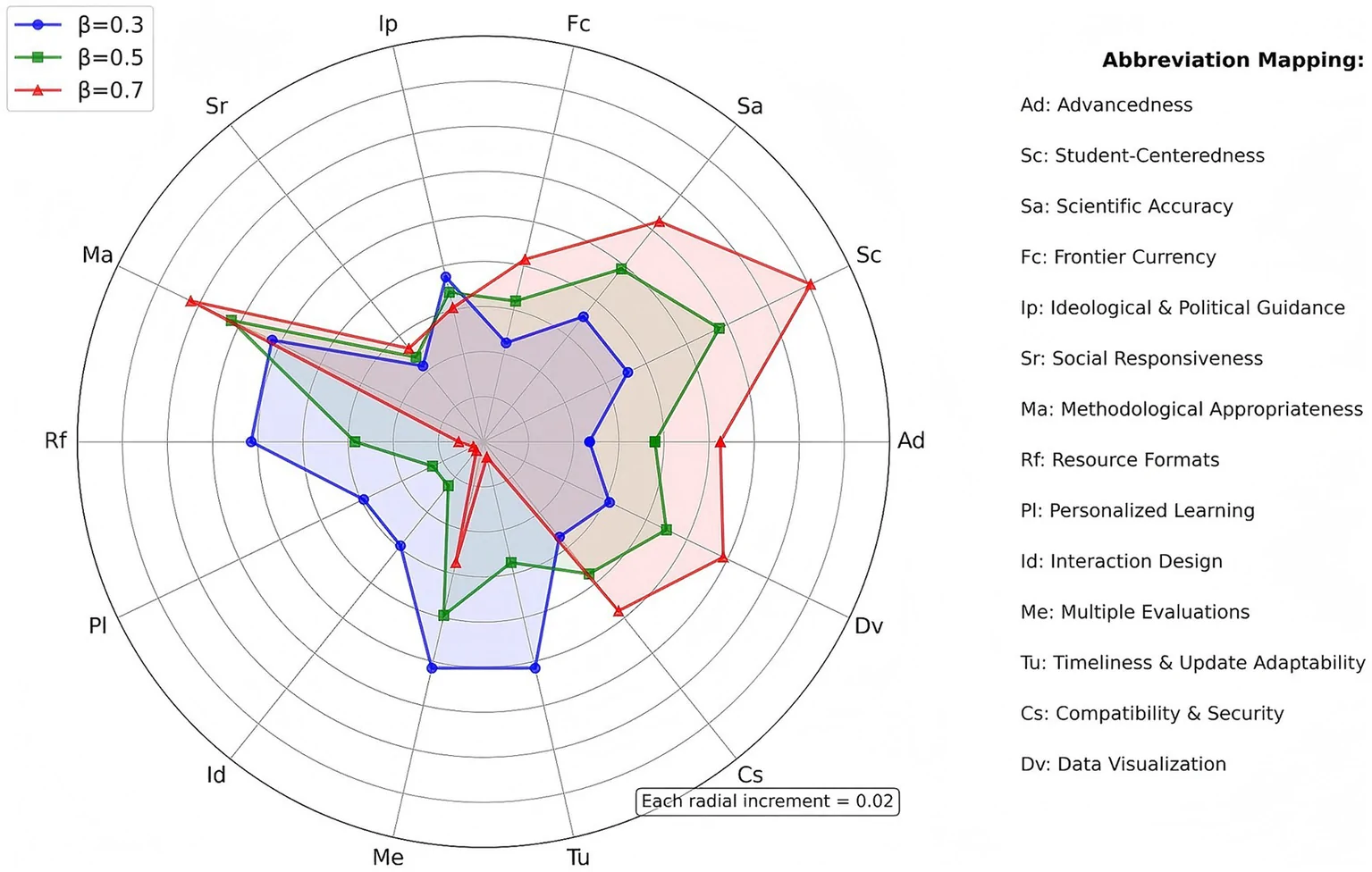
Radar chart of tertiary indicator weights under three expert-based and data-derived weighting ratios (*β* = 0.3, 0.5, 0.7). Each axis represents one tertiary indicator, and the distance from center reflects the indicator’s integrated weight under the specified *β* setting. Overlapping polygons indicate weight stability, whereas divergent vertices indicate sensitivity to the weighting ratio.

Sensitivity analysis across *γ* = 0.3, 0.5, and 0.7 indicated that the indicator rankings were highly stable: primary-dimension rank order was identical across all three settings (Spearman’s *ρ* = 1.000), and both primary-dimension and tertiary-indicator rank orders were identical (*ρ* = 1.000; exact *p* = 2.29 × 10^−11^ at the tertiary level, *n* = 14; exact *p* = 0.083 at the primary level, *n* = 4, where 0.083 is the minimum attainable two-tailed value) (Supplementary material 14; Table S14.5). The maximum relative weight deviation was 1.09%, and no indicator crossed the 0.06 integrated-weight threshold across the tested range. No rank changes were observed at any *γ* setting (Supplementary material 10).

### Analysis of the four-quadrant prioritization matrix

Figure 4 plots the 14 tertiary indicators against the two pre-specified thresholds (integrated weight = 0.06, measurability = 4.0). Seven indicators fell into Quadrant I (high weight, high measurability) — Student-Centeredness, Updated Content and Frontier Knowledge, Scientific Accuracy, Methodological Appropriateness, Interaction Design, Professional Ethics and Values Education, and Resource Formats — and can be adopted directly in standardized evaluation. Two indicators, Data Visualization and Compatibility and Security, fell into Quadrant II (high weight, low measurability): although highly valued by experts, neither reached the measurability threshold, indicating that reliable automated assessment tools are not yet available and require targeted development. No indicator fell into Quadrant III. The remaining five — Advancedness, Social Responsiveness, Multiple Evaluations, Personalized Learning, and Timeliness and Update Adaptability — fell into Quadrant IV (low weight, high measurability) and serve a supplementary role. Integrated weights ranged from 0.032 to 0.136 (median = 0.070, IQR = 0.049–0.093), so the 0.06 threshold lay between the median and the lower quartile. Four indicators lay close to a cut-off (Compatibility and Security, measurability = 3.99; Personalized Learning, 4.01; Resource Formats, weight = 0.060; Advancedness, 0.059), and their quadrant assignments should be read as boundary-sensitive. Varying the measurability cut-off from 3.8 to 4.2 left the overall structure stable: at least 6 of the 7 Quadrant I indicators were retained and no indicator moved more than one quadrant; at cut-offs above 4.0, Resource Formats crossed from Quadrant I to II, while Social Responsiveness, Personalized Learning, and Timeliness and Update Adaptability moved from Quadrant IV to III (Supplementary material 8; Section 8.2).

**Figure 4:**
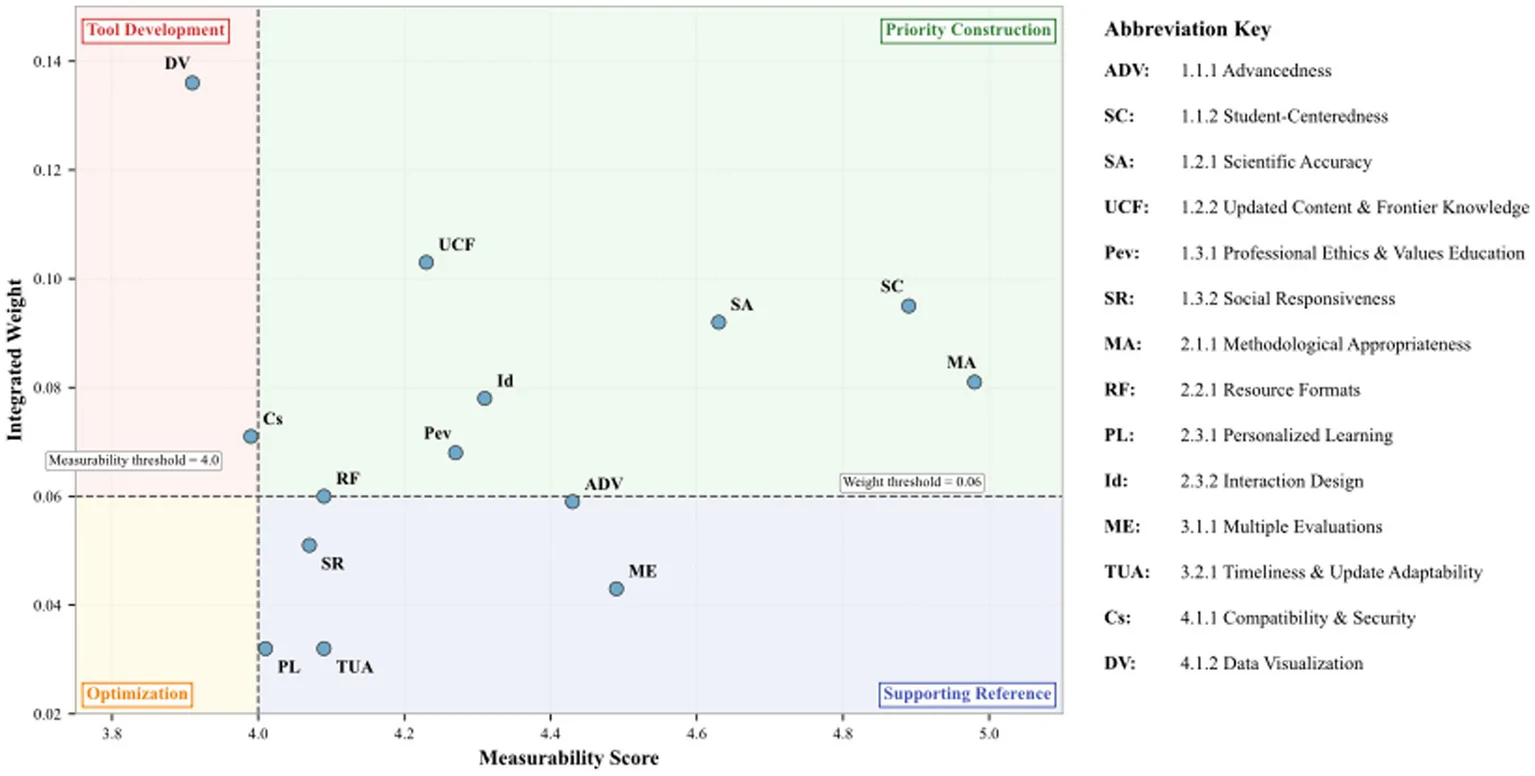
Two-dimensional partitioning matrix of 14 tertiary indicators plotted against integrated weight and measurability score. This visualization stratifies indicators into four zones to formulate differentiated implementation strategies for DLR quality evaluation. Vertical dashed line: measurability threshold = 4.0; horizontal dashed line: weight threshold = 0.06.

### Reliability and validity validation results

#### Reliability results

A total of 343 questionnaires were distributed, of which 326 were returned and 309 were retained after exclusion of 17 incomplete or invalid responses, yielding an effective response rate of 90.1%. A full demographic breakdown of the 309 validation students is provided in Supplementary material 3.

In summary, participants were distributed across clinical medicine (156 students, 50.5%) and preventive medicine (153 students, 49.5%) programs, and across Years 2–4 (Year 2: 80 students, 25.9%; Year 3: 76 students, 24.6%; Year 4: 153 students, 49.5%).

The overall Cronbach’s alpha coefficient for the 14-item evaluation system was 0.987. Cronbach’s *α* values for the four primary dimensions ranged from 0.939 to 0.970 (Content Quality: 0.970; Instructional Design: 0.963; Evaluation and Feedback: 0.939; Platform Functionality: 0.949), all above 0.90. Given the very high overall *α* and the multidimensional nature of the framework, we examined corrected item-total correlations (CITC) and alpha-if-item-deleted statistics to assess potential item redundancy (Supplementary material 11). All 14 items showed CITC > 0.88 (minimum = 0.890 for Professional Ethics and Values Education), and removal of any single item reduced the overall alpha by no more than 0.0013 (*α* if deleted: 0.9861–0.9869), suggesting that while items are highly correlated, each contributes non-redundant information. An exploratory factor analysis (principal axis factoring with varimax rotation) was conducted to examine the underlying factor structure; Bartlett’s test of sphericity was significant (*χ*^2^ = 7177.68, *p* < 0.001) and the Kaiser-Meyer-Olkin measure was 0.973, indicating excellent sampling adequacy. A 4-factor solution emerged, explaining 88.76% of total variance (27.40%, 27.09%, 19.30%, and 14.97% for Factors 1–4, respectively). All items showed primary loadings ≥0.51 on their dominant factor, and no cross-loading exceeded that primary loading. Of note, the empirical factor boundaries diverged partially from the *a priori* dimensions—platform, instructional-design, and evaluation items loaded on a common factor, and Content Quality split across two factors—consistent with the very high inter-item correlations (see CITC above). The EFA therefore supports the statistical coherence of the item set while indicating that the four-dimension structure should be regarded as a conceptual (expert-derived) rather than purely empirical grouping.

For inter-rater reliability, ICC(2,1) was computed for each course with students treated as raters and the 14 tertiary indicators as targets. Per-course ICC estimates ranged from −0.005 to 0.008, with a pooled ICC(2,1) of 0.000 (95% CI: [−0.0003, 0.0004]) across all ten courses, −0.000 across the five clinical-medicine courses (95% CI: [−0.0019, 0.0014]), and 0.001 across the five preventive-medicine courses (95% CI: [−0.0052, 0.0064]) (Supplementary material 12). The near-zero ICC values indicate substantial inter-student variability in absolute scoring levels, likely reflecting individual differences in rating leniency rather than disagreement on relative indicator importance.

#### Validity results

Content Validity: The scale-level content validity index (S-CVI) was 0.951, exceeding the recommended threshold of 0.80, with item-level CVIs (I-CVIs) ranging from 0.769 to 1.000. Thirteen of 14 indicators met the I-CVI ≥ 0.78 threshold. Under a pre-specified exception rule, an item below 0.78 was retained if it met the Round 2 consensus criteria (*M* > 3.50 and CV < 0.25) and was judged by the research team to address a non-redundant, educationally critical construct. The single below-threshold indicator, Compatibility and Security (I-CVI = 0.769; 20/26 experts), satisfied these conditions based on its acceptable consensus (CV = 0.19), above-floor importance mean (*M* = 4.19), and qualitative expert feedback identifying platform security as indispensable for digital learning resource deployment ((23); Supplementary material 8; Table S8.3). Content validity evidence is presented as one component of instrument validity and should be interpreted alongside reliability and structural validity findings.

## Discussion

This study developed and validated a set of quality evaluation indicators for medical digital learning resources using a mixed-methods approach. The final system comprises 4 primary dimensions, 9 secondary indicators, and 14 tertiary indicators, with high internal consistency and satisfactory content validity; absolute-agreement inter-rater reliability was near zero, reflecting between-student variability in scoring levels rather than disagreement on relative indicator importance.

The core methodological feature is the integration of an expert-based and data-derived weighting model (AHP-EWM) with a measurability correction mechanism, which attempts to address the gap between educational importance and operational feasibility in DLR evaluation.

### Principal findings

First, Content Quality received the highest integrated weight (0.469) among the four primary dimensions, reflecting that scientific accuracy is a universal core priority for high-quality professional education DLRs.

This finding is consistent with competency-based medical education (CBME) principles, which position evidence-based, clinically aligned foundational knowledge as a prerequisite for clinical performance (27, 28). Unlike generic OER rubrics that prioritize technical accessibility and licensing (3, 5), our results suggest that in medical education, content validity should be given priority over technical features when evaluating DLR quality. While Chan et al. (29) cataloged existing rating tools for medical OERs and Cook and Ellaway (6) outlined a process-oriented evaluation framework for TEL, neither incorporated empirical weighting nor addressed the gap between educational importance and operational feasibility—is a distinction our framework achieves through the measurability correction mechanism and four-quadrant prioritization strategy.

Second, Data Visualization (final weight = 0.136) and Updated Content and Frontier Knowledge (final weight = 0.103) were the two highest-weighted tertiary indicators. This finding suggests that pedagogical design features, particularly the alignment of teaching methods with active learning strategies, carry substantial weight in DLR quality evaluation. While our study did not directly compare the relative impact of technology integration versus pedagogical design on learning outcomes, the weight distribution indicates that experts assigned greater importance to how resources support competency-oriented learning than to the technical sophistication of the resources themselves.

Third, the measurability correction mechanism identified two indicators (Data Visualization and Compatibility and Security) that, despite being valued by experts, currently lack reliable objective measurement tools. This finding highlights a practical challenge: some educationally important aspects of DLR quality remain difficult to assess through automated platform data alone. The four-quadrant matrix provides a transparent classification that distinguishes between indicators suitable for direct implementation and those requiring further methodological development.

### Transferability and broader implications

A key strength of the proposed framework is its potential transferability across health professions education. Its methodological workflow and core dimensional structure may be generalizable to health-related disciplines, with modular calibration mechanisms to accommodate different professional standards; however, formal cross-disciplinary validation remains to be conducted.

First, the full methodological pipeline is applicable across health professions. The three-stage procedure—modified Delphi consensus for indicator construction, AHP-EWM integrated weighting, and measurability correction with a four-quadrant matrix—addresses common challenges in DLR evaluation for all health disciplines: subjective weighting bias, the importance-measurability disconnect, and the lack of stratified implementation strategies. It has the potential to be extended to nursing, pharmacy, public health, and other health education fields, subject to discipline-specific re-calibration and validation.

Second, the framework adopts a two-layer structure: four core primary dimensions (Content Quality, Instructional Design, Evaluation and Feedback, Platform Functionality) capture shared attributes of high-quality DLRs in health education, while discipline-specific calibration is implemented at the tertiary indicator level. Operational definitions, professional ethics requirements, industry standard alignment and weight distributions can be adjusted via expert consultation to match different professional contexts.

Third, the four-quadrant prioritization strategy provides an implementation roadmap adaptable across health education institutions. This phased approach reduces the cost of DLR quality governance and offers direct practical guidance for teaching administrators in medical and public health schools.

### Methodological considerations

Our study addressed three limitations in existing DLR evaluation research. First, the framework’s medical specificity and its transferability are two sides of the same coin rather than competing claims. The high-stakes, standards-intensive nature of medical education provides a rigorous test of the framework’s robustness rather than limiting its scope, and this dual character operates at three levels. At the content definition level, the core connotation of each indicator is universal, while operational definitions are anchored in disciplinary contexts: in this study, Scientific Accuracy was calibrated to medical curriculum standards and clinical practice guidelines, Methodological Appropriateness was aligned with PBL, CBL, and TBL pedagogies predominant in medical education, and Updated Content and Frontier Knowledge demands currency with medical advancements where outdated content can compromise patient safety. At the weight distribution level, Content Quality received the highest integrated weight (0.469), far exceeding approximately 12.5% per dimension in the equally weighted ROER framework (5); this elevated weight reflects the amplified importance of accuracy in high-stakes disciplines where scientific accuracy is a patient safety imperative (27, 28)—not a uniquely medical feature but a pattern consistent with quality priorities across higher education. A quantitative comparison of the present framework against three representative DLR evaluation models (ROER, TEL, and (30)) is provided in Supplementary material 13, highlighting the unique combination of integrated weighting, measurability correction, and four-quadrant prioritization in the present work. At the standards alignment level, the Professional Ethics and Values (Ideological and Political Guidance) dimension maps to generic professional competency frameworks; its alignment with the “ethical behavior and professionalism” requirements of WFME (31) and the “Professional” role in CanMEDS 2015, along with Social Responsiveness aligning with WFME’s social accountability (31) and transformative education goals (28, 32), represents a context-specific instantiation of a universal design principle. That the framework performed reliably under the strict constraints of medical education confirms its methodological robustness; whether this translates to effective cross-disciplinary application is examined in the following section.

Second, the dual AHP-EWM weighting model carries an important limitation regarding component independence. Because EWM weights are computed from the same Round 2 expert rating matrix used for the Delphi consensus process, they are not independent of expert judgment and do not constitute an external objective benchmark; rather, they reflect the discriminatory power of each indicator within the consensus data. Empirically, EWM weights showed substantial differentiation (0.072–0.445 at the primary level; 0.016–0.198 at the tertiary level), with Platform Functionality receiving the highest EWM weight and Evaluation and Feedback the lowest. This indicates that expert ratings exhibited greater dispersion for platform-related indicators, whereas consensus was highest for evaluation-related indicators. Unlike the case where EWM approximates equal weighting, the integrated weights reflect a meaningful contribution from both AHP and EWM components, with EWM particularly elevating the importance of platform functionality indicators. However, because both components ultimately derive from the same expert panel, the integrated weights should be interpreted as a consensus-based weighting system rather than a convergence of two fully independent information sources. Future studies could incorporate external usage or performance data as a third weighting source to strengthen component independence. Furthermore, the equal weighting coefficient (*β* = 0.5) was selected as a neutral default in the absence of empirical evidence favoring either component; sensitivity analysis (*β* = 0.3–0.7) indicated that primary-dimension rankings were stable at *β* ≥ 0.5, whereas indicator-level rankings remained highly correlated even at the extreme *β* = 0.3 setting (Spearman’s *ρ* = 0.928), suggesting that the default should be interpreted as a reasonable compromise rather than an empirically optimized value.

Third, the measurability correction mechanism represents an effort to address the gap between indicator importance and operational feasibility. By calibrating weights based on whether indicators can be assessed through objective platform data, the system attempts to provide a more practical evaluation tool. However, this mechanism is preliminary and requires further validation in diverse educational settings.

A related limitation concerns the four-quadrant thresholds: the weight cut-off of 0.06 and the measurability cut-off of 4.0 are expert-consensus heuristics rather than empirically derived values. Although the threshold sensitivity analysis indicated that quadrant assignments were broadly stable, indicators lying near either cut-off (e.g., Resource Formats and Timeliness and Update Adaptability near the weight threshold; Compatibility and Security and Personalized Learning near the measurability threshold) require contextual judgment in practical application. The *γ* = 0.5 correction coefficient was selected as a neutral default; sensitivity analysis indicated robust top-rank stability across *γ* = 0.3–0.7, but the optimal value may vary across contexts and should be calibrated in future applications.

### Practical application example

To illustrate, consider a medical school choosing between two DLRs for a Pathophysiology course: DLR A, a curriculum-aligned MOOC with limited personalization, and DLR B, a technically sophisticated virtual simulation with outdated content. Because Content Quality carries 46.9% of total weight, DLR A’s strength in scientific accuracy and Updated Content and Frontier Knowledge would yield a higher weighted score than DLR B’s technical advantages. The four-quadrant matrix further differentiates their weaknesses: DLR A’s limitation (Personalized Learning) falls in Quadrant IV (supplementary role) — measurable but low-priority, addressable through incremental platform improvements — whereas DLR B’s limitation (Updated Content and Frontier Knowledge) falls in Quadrant I (direct implementation), a high-priority gap requiring direct remediation. This analysis would direct the school to prioritize DLR A for core content delivery while supplementing it with improved personalization features, rather than adopting DLR B despite its technical sophistication.

### Limitations

This study has several limitations. First, the modified Delphi panel and empirical validation were conducted exclusively in the Chinese medical education context, which limits immediate cross-disciplinary and cross-national generalizability. The specific indicator weights and operational calibrations reflect the requirements of China’s medical education system and health strategies (31, 32). Future studies should validate the framework in additional higher education disciplines (e.g., engineering, teacher education) and international settings to further establish its transferability and refine the discipline-specific calibration mechanism.

Second, inherent limitations of the Delphi method apply. The purposive sampling strategy may have introduced selection bias, and while two rounds of consultation are standard, additional rounds might have achieved further consensus. The feedback provided to experts in Round 2, while aggregated and anonymous, carries a small risk of convergence toward majority views. Additionally, AHP consistency filtering retained 15 of 26 experts (57.7%), excluding 11 whose pairwise comparison matrices did not meet the CR < 0.10 threshold. While this threshold is standard (18), the high exclusion rate may reflect the cognitive burden of multiple pairwise comparisons and could introduce selection bias if excluded experts held different priorities.

Third, the framework was developed and validated for clinical and preventive medicine only. Application to other health professions (e.g., nursing, pharmacy) would require modular calibration of indicators and weights. Additionally, the Quadrant II indicators (Data Visualization, Compatibility and Security) remain dependent on expert review rather than fully automated assessment. The high overall Cronbach’s *α* (0.987) may also suggest redundancy among certain tertiary indicators, and future iterations could examine whether some items measure overlapping constructs.

Finally, the static nature of the current indicator system may not fully capture the rapid evolution of educational technologies, particularly the integration of generative AI tools into medical DLRs. Future iterations should address emerging constructs such as algorithmic ethics and AI-assisted learning validity. Sex and age were not collected in the student survey to preserve anonymity, limiting full demographic characterization of the validation sample.

## Conclusion

This study developed and validated a quality evaluation framework for digital learning resources using a mixed-methods approach that integrates modified Delphi consensus, AHP-EWM integrated weighting, and measurability correction. The final system comprises 4 primary dimensions, 9 secondary indicators, and 14 tertiary indicators, with high internal consistency and satisfactory content validity, while absolute-agreement inter-rater reliability was limited by between-student variability in scoring levels.

The core methodological contribution is the two-stage calibration approach that balances educational importance and operational feasibility, paired with a four-quadrant prioritization matrix for phased implementation. Validated in the Chinese medical education context, the core workflow shows potential for transferability to other higher education disciplines via discipline-specific contextual calibration, pending cross-context validation. Future research may extend validation to diverse disciplinary and international settings.

## Data Availability

The original contributions presented in the study are included in the article/Supplementary material, further inquiries can be directed to the corresponding author.

## References

[1] McCarthy AM Maor D McConney A Cavanaugh C. Digital transformation in education: critical components for leaders of system change. Soc Sci Humanit Open. (2023) 8:100479. doi: 10.1016/j.ssaho.2023.100479

[2] Felix HM Schertzer K. Mastery learning in medical simulation. In: StatPearls. Treasure Island (FL): StatPearls Publishing (2022). Available from: https://www.ncbi.nlm.nih.gov/books/NBK547741/32310378

[3] Yuan M Recker M. Not all rubrics are equal: a review of rubrics for evaluating the quality of open educational resources. Int Rev Res Open Dist Learn. (2015) 16:16–38. doi: 10.19173/irrodl.v16i5.2389

[4] Bates AW. Teaching in a Digital Age: Guidelines for Designing Teaching and Learning for a Digital Age. 2nd ed.Tony Bates Associates Ltd. (2019) 1–16.

[5] Achieve Inc. (2011). Rubrics for evaluating open education resource (OER) objects. Available online at: https://www.achieve.org/files/AchieveOERRubrics.pdf (Accessed September 30, 2026)

[6] Cook DA Ellaway RH. Evaluating technology-enhanced learning: a comprehensive framework. Med Teach. (2015) 37:961–70. doi: 10.3109/0142159X.2015.1009024, 25782599

[7] He J Ma T. A deep learning-oriented evaluation model and index system for blended learning in Chinese higher vocational education. Innov Educ Teach Int. (2025). doi: 10.1080/14703297.2025.2569510

[8] Zakeri S Konstantas D Chatterjee P Zavadskas EK. Soft cluster-rectangle method for eliciting criteria weights in multi-criteria decision-making. Sci Rep. (2025) 15:284. doi: 10.1038/s41598-024-81027-4, 39747895 PMC11755851

[9] Potter A Munsch C Watson E Hopkins E Kitromili S O’Neill IC . Identifying research priorities in digital education for health care: umbrella review and modified Delphi method study. J Med Internet Res. (2025) 27:e66157. doi: 10.2196/66157 PMC11888089 39969988

[10] Sun X Fu Y Wang W. Optimal weighting method for subjective and objective integration with subjective consultation constraints. Syst. Eng. Electron. (2025) 47:3004–10.

[11] Zhao T Yu L Jiang X Yang S Zhang X Zhang M . Construction of a public health practice teaching quality evaluation system based on the CIPP model using the analytic hierarchy process (AHP) and entropy weight method. BMC Med Educ. (2025) 25:368. doi: 10.1186/s12909-025-06943-z, 40075366 PMC11899688

[12] Diamond IR Grant RC Feldman BM Pencharz PB Ling SC Moore AM . Defining consensus: a systematic review recommends methodologic criteria for reporting of Delphi studies. J Clin Epidemiol. (2014) 67:401–9. doi: 10.1016/j.jclinepi.2013.12.002, 24581294

[13] Artsi Y Sorin V Glicksberg B Korfiatis P Thomas DC Nadkarni GN . AI-generated videos in medical education: systematic review. BMJ Open Qual. (2025) 14:e003704. doi: 10.1136/bmjoq-2025-003704, 41429451 PMC12742227

[14] Zhou J Zhang J Wan R Cui X Liu Q Guo H . Integrating AI into clinical education: evaluating general practice trainees’ proficiency in distinguishing AI-generated hallucinations and impacting factors. BMC Med Educ. (2025) 25:406. doi: 10.1186/s12909-025-06916-2, 40108629 PMC11924592

[15] Saaty TL. The analytic hierarchy process: planning, priority setting, resource allocation. New York: McGraw-Hil (1980)

[16] Ng IKS Mok SF Teo D. Competency in medical training: current concepts, assessment modalities, and practical challenges. Postgrad Med J. (2024) 100:1–8. doi: 10.1093/postmj/qgae023, 38376156

[17] von der Gracht HA. Consensus measurement in Delphi studies: review and implications for future quality assurance. Technol Forecast Soc Change. (2012) 79:1525–36. doi: 10.1016/j.techfore.2012.04.013

[18] Saaty TL. How to make a decision: the analytic hierarchy process. Interfaces. (1994) 24:19–43. doi: 10.1287/inte.24.6.19

[19] Al-Aomar R. A combined AHP-entropy method for deriving subjective and objective criteria weights. Int J Ind Eng Theory Appl Pract. (2010) 17:12–24. doi: 10.23055/ijietap.2010.17.1.330

[20] Niederberger M Köberich S members of the DeWiss Network. Coming to consensus: the Delphi technique. Eur J Cardiovasc Nurs. (2021) 20:692–5. doi: 10.1093/eurjcn/zvab059 34245253

[21] Hasson F Keeney S McKenna H. Research guidelines for the Delphi survey technique. J Adv Nurs. (2000) 32:1008–15. doi: 10.1046/j.1365-2648.2000.t01-1-01567.x 11095242

[22] Zhang X Fang S Jin Y Huang Y Wang S Wang J . Comprehensive performance evaluation of valuable medical equipment based on cloud modelling and combined weighting methodologies. Eng Appl Artif Intell. (2025) 154:110969. doi: 10.1016/j.engappai.2025.110969

[23] Polit DF Beck CT. The content validity index: are you sure you know what’s being reported? Critique and recommendations. Res Nurs Health. (2006) 29:489–97. doi: 10.1002/nur.20147 16977646

[24] Koo TK Li MY. A guideline of selecting and reporting intraclass correlation coefficients for reliability research. J Chiropr Med. (2016) 15:155–63. doi: 10.1016/j.jcm.2016.02.012, 27330520 PMC4913118

[25] Shrout PE Fleiss JL. Intraclass correlations: uses in assessing rater reliability. Psychol Bull. (1979) 86:420–8. doi: 10.1037/0033-2909.86.2.420, 18839484

[26] Tavakol M Dennick R. Making sense of Cronbach’s alpha. Int J Med Educ. (2011) 2:53–5. doi: 10.5116/ijme.4dfb.8dfd, 28029643 PMC4205511

[27] Frank JR Snell L Sherbino J. CanMEDS 2015 Physician Competency Framework.Royal College of Physicians and Surgeons of Canada (2015).

[28] Frenk J Chen L Bhutta ZA Cohen J Crisp N Evans T . Health professionals for a new century: transforming education to strengthen health systems in an interdependent world. Lancet. (2010) 376:1923–58. doi: 10.1016/S0140-6736(10)61854-5, 21112623

[29] Chan TM Trueger NS Maggio LA Ting DK Sherbino J Thoma B. A user’s guide to open educational resources in medical education. Cureus. (2025) 17:e93394. doi: 10.7759/cureus.93394, 41164038 PMC12560063

[30] Luo R Huang R Shi G. Development and validation of an LMOOC quality evaluation scale: a large-scale mixed-method approach. ReCALL. (2025) 38:4–21. doi: 10.1017/S0958344025000072

[31] World Federation for Medical Education (2020). Basic medical education: WFME global standards for quality improvement (2020 revision). Available online at: https://wfme.org/wp-content/uploads/2020/12/WFME-BME-Standards-2020.pdf (Accessed September 30, 2026)

[32] World Federation for Medical Education (2021). WFME standards for distributed and distance learning in medical education. Available online at: https://wfme.org/download/wfme-standards-for-distributed-and-distance-learning-in-medical-education-2021/ (Accessed September 30, 2026)

