# Supplementary material for: Development and validation of a digital learning resource quality evaluation framework: a mixed-methods study in health professions education
Source: Front Public Health. 2026 Oct 14;14:1958724. doi: 10.3389/fpubh.2026.1958724 (PMC13715655; doi:10.3389/fpubh.2026.1958724)
Supplement: Supplementary file 1 [file Table_1.DOCX]

**Supplementary Material 1: PRISMA Flow Diagram**

Figure S1.1. PRISMA 2020 flow diagram for the literature-search component of the mixed-methods study.

**Step 1 – Systematic literature search.** Searches were conducted in Web of Science Core Collection, OpenAlex, PubMed, and CNKI (January 2019–December 2024). Three a priori concept blocks—resource type (4 terms), educational context (3 terms), and evaluation focus (4 terms)—were fully crossed to yield 48 search strings, executed separately in each database (524 WoS, 319 OpenAlex, 314 PubMed, 5 CNKI; pooled and de-duplicated).

**Step 2 – Study selection.** Of 1,162 records, 72 duplicates were removed; 1,090 underwent title screening (579 excluded), 511 abstract screening (238 excluded by two independent reviewers), and 273 full-text assessment (25 excluded: no structured framework n=3, out of scope n=2, no extractable dimensions n=12, non-peer-reviewed/non-English or Chinese n=8). Ultimately, 248 studies were included for indicator extraction.

**Step 3 – Indicator pool construction.** Two researchers independently extracted indicators and synthesised a preliminary dimensional structure, supplemented by medicine-specific indicators from stakeholder (n=15) and medical-student (n=50) focus groups. The resulting pool comprised 5 primary dimensions, 11 secondary indicators, and 21 tertiary indicators.

*(Full search string list in Supplementary Material 1; focus group interview guides in Supplementary Material 2.)*
