# Supplementary material for: Development and validation of a digital learning resource quality evaluation framework: a mixed-methods study in health professions education
Source: Front Public Health. 2026 Oct 14;14:1958724. doi: 10.3389/fpubh.2026.1958724 (PMC13715655; doi:10.3389/fpubh.2026.1958724)
Supplement: Supplementary file 2 [file Table_10.docx]

**Supplementary Material 10: Sensitivity Analysis of the Measurability Correction Coefficient (γ)**

Table S10.1 Tertiary indicator final weights under γ = 0.3, 0.5, and 0.7.

| **Indicator** | **W_int_** | **Ms** | **γ=0.3** | **Rank** | **γ=0.5** | **Rank** | **γ=0.7** | **Rank** | **Δrank** |
| --- | --- | --- | --- | --- | --- | --- | --- | --- | --- |
| 1.1.1 Advancedness | 0.059 | 4.965 | 0.059 | 9.5 | 0.059 | 9.5 | 0.059 | 9.5 | 0 |
| 1.1.2 Student-Centeredness | 0.095 | 4.231 | 0.095 | 3 | 0.095 | 3 | 0.095 | 3 | 0 |
| 1.2.1 Scientific Accuracy | 0.092 | 4.081 | 0.092 | 4 | 0.092 | 4 | 0.093 | 4 | 0 |
| 1.2.2 Updated Content & Frontier Knowledge | 0.103 | 4.077 | 0.104 | 2 | 0.103 | 2 | 0.103 | 2 | 0 |
| 1.3.1 Professional Ethics & Values Education | 0.068 | 4.427 | 0.068 | 8 | 0.068 | 8 | 0.068 | 8 | 0 |
| 1.3.2 Social Responsiveness | 0.051 | 4.886 | 0.051 | 11 | 0.051 | 11 | 0.051 | 11 | 0 |
| 2.1.1 Methodological Appropriateness | 0.081 | 4.631 | 0.081 | 5 | 0.081 | 5 | 0.081 | 5 | 0 |
| 2.2.1 Resource Formats | 0.06 | 4.232 | 0.059 | 9.5 | 0.059 | 9.5 | 0.059 | 9.5 | 0 |
| 2.3.1 Personalized Learning | 0.032 | 4.267 | 0.032 | 13.5 | 0.032 | 13.5 | 0.032 | 13.5 | 0 |
| 2.3.2 Interaction Design | 0.078 | 4.066 | 0.078 | 6 | 0.078 | 6 | 0.078 | 6 | 0 |
| 3.1.1 Multiple Evaluations | 0.043 | 4.982 | 0.043 | 12 | 0.043 | 12 | 0.043 | 12 | 0 |
| 3.2.1 Timeliness & Update Adaptability | 0.032 | 4.090 | 0.032 | 13.5 | 0.032 | 13.5 | 0.032 | 13.5 | 0 |
| 4.1.1 Compatibility & Security | 0.071 | 4.011 | 0.070 | 7 | 0.070 | 7 | 0.070 | 7 | 0 |
| 4.1.2 Data Visualization | 0.136 | 4.313 | 0.135 | 1 | 0.135 | 1 | 0.135 | 1 | 0 |

Note. W_final_=W_int_×[1+γ×(*M*s_normalized_−0.5)], where W_int_ is the integrated AHP-EWM weight and *M*s_normalized_ is the min–max normalised measurability score (range 0–1). Rank = 1 indicates the highest weight. Δrank = maximum rank change across γ = 0.3 and γ = 0.7 relative to the γ = 0.5 baseline.

Table S10.2. Primary dimension aggregated weights under γ = 0.3, 0.5, and 0.7.

| **Primary Dimension** | **γ=0.3** | **Rank** | **γ=0.5** | **Rank** | **γ=0.7** | **Rank** |
| --- | --- | --- | --- | --- | --- | --- |
| Content Quality | 0.4716 | 1 | 0.4743 | 1 | 0.4769 | 1 |
| Instructional Design | 0.2525 | 2 | 0.2536 | 2 | 0.2548 | 2 |
| Platform Functionality | 0.2011 | 3 | 0.1973 | 3 | 0.1935 | 3 |
| Evaluation & Feedback | 0.0749 | 4 | 0.0748 | 4 | 0.0748 | 4 |

**Summary Statistics**

- Spearman rank correlation (tertiary, n=14): γ=0.3 vs 0.5: ρ = 1.000 (P < 0.001); γ=0.7 vs 0.5: ρ = 1.000 (P < 0.001).
- Spearman rank correlation (primary, n=4): ρ = 1.000 for both comparisons; rank order identical across all γ values.
- Maximum absolute weight deviation: 0.0010 (Updated Content & Frontier Knowledge, γ=0.3 vs 0.5; Scientific Accuracy, γ=0.7 vs 0.5).
- Maximum relative weight deviation: 1.09% (Scientific Accuracy, γ=0.7 vs 0.5).
- Mean absolute deviation: 0.0001; mean relative deviation: 0.07%.
- No indicator crossed the 0.06 integrated-weight threshold across γ = 0.3–0.7.
- No rank changes were observed at any γ setting; all 14 tertiary indicators retained identical rank order across the tested range.
