# Supplementary material for: Development and validation of a digital learning resource quality evaluation framework: a mixed-methods study in health professions education
Source: Front Public Health. 2026 Oct 14;14:1958724. doi: 10.3389/fpubh.2026.1958724 (PMC13715655; doi:10.3389/fpubh.2026.1958724)
Supplement: Supplementary file 3 [file Table_11.docx]

**Supplementary Material 11: Item Analysis (CITC, Alpha-if-Deleted) and Exploratory Factor Analysis**

Table S11.1 Item-level descriptive statistics, corrected item-total correlations (CITC), and Cronbach's α if item deleted (N = 309)

| Dimension | Indicator | Mean | SD | CITC | Cronbach's α if item deleted |
| --- | --- | --- | --- | --- | --- |
| Content Quality | Educational Philosophy—Advancedness | 4.307 | 0.768 | 0.9138 | 0.9865 |
| Content Quality | Educational Philosophy—Student-Centeredness | 4.304 | 0.805 | 0.9119 | 0.9865 |
| Content Quality | Content Scientificity—Scientific Accuracy | 4.311 | 0.773 | 0.8969 | 0.9867 |
| Content Quality | Content Scientificity—Updated Content & Frontier Knowledge | 4.265 | 0.786 | 0.8989 | 0.9867 |
| Content Quality | Professional Ethics & Values Education | 4.301 | 0.788 | 0.8897 | 0.9869 |
| Content Quality | Social Responsiveness | 4.282 | 0.795 | 0.9128 | 0.9865 |
| Instructional Design | Teaching Method—Methodological Appropriateness | 4.317 | 0.762 | 0.904 | 0.9866 |
| Instructional Design | Resource Diversity—Resource Formats | 4.307 | 0.751 | 0.9249 | 0.9863 |
| Instructional Design | Learning Support—Personalized Learning | 4.294 | 0.769 | 0.9256 | 0.9863 |
| Instructional Design | Learning Support—Interaction Design | 4.291 | 0.76 | 0.9327 | 0.9862 |
| Evaluation & Feedback | Learning Assessment—Multiple Evaluations | 4.307 | 0.751 | 0.9199 | 0.9864 |
| Evaluation & Feedback | Application Feedback—Timeliness & Update Adaptability | 4.282 | 0.753 | 0.9398 | 0.9861 |
| Platform Functionality | Interactive Experience—Compatibility & Security | 4.285 | 0.766 | 0.9129 | 0.9865 |
| Platform Functionality | Interactive Experience—Data Visualization | 4.301 | 0.762 | 0.9317 | 0.9862 |

Note. CITC = corrected item-total correlation; α if item deleted = Cronbach's alpha resulting from removing that item.

Table S11.2 Cronbach's α by primary dimension

| Dimension | Number of items | Cronbach's α |
| --- | --- | --- |
| Content Quality | 6 | 0.9703 |
| Instructional Design | 4 | 0.9632 |
| Evaluation & Feedback | 2 | 0.9394 |
| Platform Functionality | 2 | 0.949 |
| Overall (14 items) | 14 | 0.9874 |

Table S11.3 Exploratory factor analysis factor loadings (varimax-rotated, principal axis factoring)

| Dimension | Indicator | Factor 1 | Factor 2 | Factor 3 | Factor 4 |
| --- | --- | --- | --- | --- | --- |
| Content Quality | Educational Philosophy—Advancedness | 0.411 | 0.726 | 0.344 | 0.326 |
| Content Quality | Educational Philosophy—Student-Centeredness | 0.424 | 0.649 | 0.378 | 0.362 |
| Content Quality | Content Scientificity—Scientific Accuracy | 0.395 | 0.653 | 0.426 | 0.305 |
| Content Quality | Content Scientificity—Updated Content & Frontier Knowledge | 0.445 | 0.616 | 0.409 | 0.308 |
| Content Quality | Professional Ethics & Values Education | 0.42 | 0.437 | 0.583 | 0.367 |
| Content Quality | Social Responsiveness | 0.443 | 0.443 | 0.686 | 0.278 |
| Instructional Design | Teaching Method—Methodological Appropriateness | 0.472 | 0.451 | 0.325 | 0.617 |
| Instructional Design | Resource Diversity—Resource Formats | 0.558 | 0.487 | 0.385 | 0.418 |
| Instructional Design | Learning Support—Personalized Learning | 0.619 | 0.472 | 0.348 | 0.402 |
| Instructional Design | Learning Support—Interaction Design | 0.627 | 0.476 | 0.427 | 0.313 |
| Evaluation & Feedback | Learning Assessment—Multiple Evaluations | 0.519 | 0.41 | 0.439 | 0.506 |
| Evaluation & Feedback | Application Feedback—Timeliness & Update Adaptability | 0.546 | 0.496 | 0.444 | 0.391 |
| Platform Functionality | Interactive Experience—Compatibility & Security | 0.623 | 0.412 | 0.422 | 0.362 |
| Platform Functionality | Interactive Experience—Data Visualization | 0.702 | 0.423 | 0.397 | 0.32 |
|  | Variance explained (%) | 27.4 | 27.09 | 19.3 | 14.97 |
|  | Cumulative variance (%) | 27.4 | 54.49 | 73.79 | 88.76 |

Table S11.4 Exploratory factor analysis summary statistics

| Statistic | Value |
| --- | --- |
| Bartlett χ² | 7177.68 |
| Bartlett P-value | <0.001 |
| KMO | 0.9733 |
| Number of factors | 4 |
| Extraction method | Principal Axis Factoring |
| Rotation method | Varimax |
| Cumulative variance explained (%) | 88.76 |
