# Supplementary material for: Development and validation of a digital learning resource quality evaluation framework: a mixed-methods study in health professions education
Source: Front Public Health. 2026 Oct 14;14:1958724. doi: 10.3389/fpubh.2026.1958724 (PMC13715655; doi:10.3389/fpubh.2026.1958724)
Supplement: Supplementary file 4 [file Table_12.docx]

**Supplementary material 12:**

Table S12.1 Per-Course Intraclass Correlation Coefficients for the Student Validation Sample

| Course | Course Type | n_students | ICC(2,1) | 95% CI Lower | 95% CI Upper | F | df1 | df2 | P-value |
| --- | --- | --- | --- | --- | --- | --- | --- | --- | --- |
| Clinical Skills | Clinical | 35 | -0.0019 | -0.0037 | 0.0042 | 0.666 | 13 | 442 | 0.7970 |
| Diagnostic Imaging | Clinical | 13 | 0.0005 | -0.0008 | 0.0049 | 1.251 | 13 | 156 | 0.2488 |
| Diagnostics | Clinical | 43 | 0.001 | -0.0021 | 0.0111 | 1.186 | 13 | 546 | 0.2856 |
| Internal Medicine | Clinical | 34 | 0.0007 | -0.0005 | 0.0045 | 1.372 | 13 | 429 | 0.1692 |
| Surgery | Clinical | 31 | -0.0014 | -0.0041 | 0.0077 | 0.805 | 13 | 390 | 0.6547 |
| Epidemiology | Preventive | 29 | -0.0052 | -0.0072 | 0.0012 | 0.433 | 13 | 364 | 0.9572 |
| Medical Statistics | Preventive | 32 | -0.0012 | -0.0034 | 0.0061 | 0.794 | 13 | 403 | 0.6671 |
| Sanitary Chemistry | Preventive | 32 | 0.0011 | -0.0008 | 0.0074 | 1.366 | 13 | 403 | 0.1726 |
| Sanitary Microbiology | Preventive | 27 | 0.0076 | 0.0014 | 0.0277 | 2.464 | 13 | 338 | 0.0033 |
| Toxicology | Preventive | 33 | 0.0007 | -0.0025 | 0.0112 | 1.114 | 13 | 416 | 0.3448 |
| Clinical (pooled) | Clinical | 156 | -0.0002 | -0.0019 | 0.0014 |  |  |  |  |
| Preventive (pooled) | Preventive | 153 | 0.0006 | -0.0052 | 0.0064 |  |  |  |  |
| All courses (pooled) | All | 309 | -0.0001 | -0.0003 | 0.0004 | 0.706 | 13 | 4004 | 0.7593 |
