# Supplementary material for: Development and validation of a digital learning resource quality evaluation framework: a mixed-methods study in health professions education
Source: Front Public Health. 2026 Oct 14;14:1958724. doi: 10.3389/fpubh.2026.1958724 (PMC13715655; doi:10.3389/fpubh.2026.1958724)
Supplement: Supplementary file 5 [file Table_13.docx]

**Supplementary material 13: Comparison of the present framework with representative DLRTEL evaluation models**

Table S13.1 Comparison of the present framework with representative DLR/TEL evaluation models

| **Dimension** | **Present Study** | **ROER Rubric (Achieve, 2011)** | **TEL Framework (Cook & Ellaway, 2015)** | **LMOOC Quality Evaluation Scale (**Luo, R.et al. , **2025)** |
| --- | --- | --- | --- | --- |
| **Primary dimensions** | 4 (Content Quality, Instructional Design, Evaluation & Feedback, Platform Functionality) | 8 rubrics (Alignment, Explanation, Utility, Assessment, Interactivity, Practice Exercises, Deeper Learning, Accessibility) | Process-oriented framework (no fixed indicator dimensions; organized around evaluation questions and outcomes) | 4 factors (Instructional Design, L2 Teachers' Competence, Teaching Implementation, Technical Support) |
| **Number of indicators/items** | 14 tertiary indicators | 8 rubrics with multiple criteria | Not specified (flexible framework) | 26 items |
| **Weighting method** | AHP-EWM integrated weighting (β = 0.5) | Equal weighting (no explicit weighting) | No explicit weighting | Equal weighting (factor scores computed as means) |
| **Measurability correction** | Yes (γ-adjusted final weights) | No | No | No |
| **Prioritization strategy** | Yes (four-quadrant matrix) | No | No | No |
| **Sensitivity analysis** | Yes (β, γ, threshold, AHP consistency) | No | No | No (reported EFA/CFA fit indices) |
| **Disciplinary focus** | Medical and public health education | General OER (K-12 and higher education) | Medical education | Language education (LMOOCs) |
| **Validation approach** | Delphi (26 experts, 2 rounds) + psychometric validation (309 students, α, ICC, EFA) | Expert review and field testing | Theoretical framework development + learner/instructor perception instruments | Mixed-method: literature review + focus groups + EFA + CFA (large-scale learner sample) |

Note. ROER = Open Educational Resource Rubric; TEL = Technology-Enhanced Learning; LMOOC = Language Massive Open Online Course; EFA = Exploratory Factor Analysis; CFA = Confirmatory Factor Analysis. The Cook & Ellaway (2015) framework is a process-oriented evaluation model rather than a fixed-dimensional indicator system, and is included to represent the broader TEL evaluation landscape in medical education. The LMOOC Quality Evaluation Scale (2025) is included as a recent, psychometrically validated MOOC evaluation instrument with a fixed four-factor structure.
