# Supplementary material for: Development and validation of a digital learning resource quality evaluation framework: a mixed-methods study in health professions education
Source: Front Public Health. 2026 Oct 14;14:1958724. doi: 10.3389/fpubh.2026.1958724 (PMC13715655; doi:10.3389/fpubh.2026.1958724)
Supplement: Supplementary file 6 [file Table_14.DOCX]

**Supplementary Material 14: Additional Statistical Analyses of Expert Selection, Weighting Inputs and Rank Stability**

# **Table S14.1. Demographic comparison between retained and excluded experts.**

| Variable | Retained (n = 15) | Excluded  (n = 11) | Test statistic | P |
| --- | --- | --- | --- | --- |
| Professional field |  |  | χ² = 7.271, df = 3 | 0.064 (permutation 0.073) |
| Educational Administration | 5 | 0 |  |  |
| Medical Education Research | 5 | 2 |  |  |
| Clinical Medicine Expert | 2 | 5 |  |  |
| Teaching Expert | 3 | 4 |  |  |
| Professional title |  |  | χ² = 5.588, df = 2 | 0.061 (permutation 0.072) |
| Senior (Professor-level) | 11 | 3 |  |  |
| Associate Senior | 3 | 5 |  |  |
| Intermediate | 1 | 3 |  |  |
| Years of Work Experience | 27.20 ± 12.19 | 19.18 ± 10.92 | Mann–Whitney U = 113.5 | 0.111 |

Note. Monte Carlo permutation tests (20,000 permutations) were used for categorical variables because several expected counts were below 5; permutation P values are in parentheses. No significant differences were found (all *P* > 0.05).

# **Table S14.2. Descriptive statistics of the EWM input matrix.**

| **Statistic** | **Value** |
| --- | --- |
| Overall mean (M) | 4.59 |
| Overall standard deviation (SD) | 0.59 |
| Minimum / maximum cell value | 2/5 |
| Rating = 5 (top score) | 234 cells (64.3%) |
| Rating = 4 | 112 cells (30.8%) |
| Rating = 3 | 17 cells (4.6%) |
| Rating = 2 | 1 cell (0.3%) |
| Rating = 1 | 0 cells (0.00%) |
| Item-level mean range | 4.19 – 4.89 |
| Item-level SD range | 0.33 – 0.80 |

# **Table S14.3. Item-level descriptive statistics of the EWM input matrix.**

| Indicator | Mean | SD | Min | Max | Variance |
| --- | --- | --- | --- | --- | --- |
| AD (Advancedness) | 4.885 | 0.326 | 4 | 5 | 0.106 |
| SC (Student-Centeredness) | 4.846 | 0.368 | 4 | 5 | 0.135 |
| SA (Scientific Accuracy) | 4.769 | 0.514 | 3 | 5 | 0.265 |
| UCF (Updated Content & Frontier Knowledge) | 4.577 | 0.643 | 3 | 5 | 0.414 |
| Pev (Professional Ethics & Values Education) | 4.692 | 0.471 | 4 | 5 | 0.222 |
| SR (Social Responsiveness) | 4.577 | 0.504 | 4 | 5 | 0.254 |
| MA (Methodological Appropriateness) | 4.692 | 0.679 | 2 | 5 | 0.462 |
| RF (Resource Formats) | 4.808 | 0.402 | 4 | 5 | 0.162 |
| PL (Personalized Learning) | 4.577 | 0.578 | 3 | 5 | 0.334 |
| ID (Interaction Design) | 4.5 | 0.648 | 3 | 5 | 0.42 |
| ME (Multiple Evaluations) | 4.423 | 0.578 | 3 | 5 | 0.334 |
| TUA (Timeliness & Update Adaptability) | 4.192 | 0.801 | 3 | 5 | 0.642 |
| CS (Compatibility & Security) | 4.538 | 0.508 | 4 | 5 | 0.259 |
| DV (Data Visualization) | 4.192 | 0.694 | 3 | 5 | 0.482 |

Note. Values are mean ± SD; Min and Max are raw scores on the 5-point scale.

# **Table S14.4. Rank stability across β settings: Spearman rank correlations, exact p-values, and 95% confidence intervals versus the β = 0.5 baseline.**

| **Level** | **β** | **ρ** | **Exact P** | **95% CI** |
| --- | --- | --- | --- | --- |
| Primary (n = 4) | 0.3 | 0.8 | 0.333 | — |
| Primary (n = 4) | 0.4 | 1 | 0.083 | — |
| Primary (n = 4) | 0.6 | 1 | 0.083 | — |
| Primary (n = 4) | 0.7 | 1 | 0.083 | — |
| Tertiary (n = 14) | 0.3 | 0.928 | 1.72 × 10⁻⁶ | [0.783, 0.977] |
| Tertiary (n = 14) | 0.4 | 0.977 | 2.03 × 10⁻⁹ | [0.927, 0.993] |
| Tertiary (n = 14) | 0.6 | 0.968 | 1.45 × 10⁻⁸ | [0.899, 0.990] |
| Tertiary (n = 14) | 0.7 | 0.858 | 8.65 × 10⁻⁵ | [0.601, 0.954] |

Note. At the primary level, all 4! = 24 permutations were exactly enumerated; the minimum attainable exact two-tailed P is 0.083, so primary-level correlations are interpreted descriptively. At the tertiary level, confidence intervals were obtained by Fisher z transformation and P values by the corresponding t approximation. All ρ values are reproduced from Supplementary Material 9, Table S9.3.

# **Table S14.5. Rank stability across γ settings: Spearman rank correlations, exact p-values and 95% confidence intervals versus the γ = 0.5 baseline.**

| **Level/Comparison** | **ρ** | **95% CI** | **P Value** |
| --- | --- | --- | --- |
| Tertiary (n = 14), γ=0.3 vs 0.5 | 1.000 | [1.000, 1.000] | 2.29 × 10⁻¹¹ |
| Tertiary (n = 14), γ=0.7 vs 0.5 | 1.000 | [1.000, 1.000] | 2.29 × 10⁻¹¹ |
| Primary (n = 4), all three γ settings | 1.000 | Not reported | 0.083 |

Note. Rank order was identical to the γ = 0.5 baseline at every γ setting, at both the tertiary and primary levels; no rank changes were observed across the tested range. Where ρ = 1.000 the Fisher z transformation diverges and the confidence interval degenerates to a point, so the interval is reported as [1.000, 1.000]. At the primary level, all 4! = 24 permutations were exactly enumerated and the minimum attainable exact two-tailed P at n = 4 is 0.083; a confidence interval is not reported at this level because the Fisher z standard error is 1/√(n − 3) = 1.000 and any interval would span the full correlation range.
