# Supplementary material for: Development and validation of a digital learning resource quality evaluation framework: a mixed-methods study in health professions education
Source: Front Public Health. 2026 Oct 14;14:1958724. doi: 10.3389/fpubh.2026.1958724 (PMC13715655; doi:10.3389/fpubh.2026.1958724)
Supplement: Supplementary file 7 [file Table_2.DOCX]

**Supplementary material 2: Semi-structured focus group interview guides**

Semi-Structured Focus Group Interview Guides for the Quality Evaluation System of Medical digital learning resources

Distinguished Expert,

Thank you very much for taking the time to participate in this focus group interview. I am a member of the research team for the study Construction of a Quality Evaluation System for Digital Learning Resources in Medical Institutions.

Prior to this interview, we conducted a systematic literature search in the Web of Science and CNKI databases from 2019 to 2024, using the keywords "digital learning resources", "medical education", and "quality evaluation", to extract general dimensions for the quality evaluation of digital learning resources. The core purpose of this interview is to listen to your real experiences and core demands in the use, development, management, and learning of medical digital learning resources, to identify discipline-specific quality evaluation indicators for medical education that distinguish it from general academic disciplines, and to provide qualitative evidence for us to construct a scientific evaluation system.

This interview is fully anonymous, and all content will be used exclusively for academic research purposes. We will strictly maintain the confidentiality of the interview content, and will never disclose any personal information. There are no right or wrong answers in this interview, and any of your ideas and suggestions are extremely valuable to our research. If you have any questions during the interview, please feel free to raise them at any time. Could you please confirm that you have fully understood the above statements, and whether you agree to the audio recording of this interview?

Thank you for your support!

**Outline 1: Interview Outline for Stakeholder Experts**

**Target Population**: Medical education researchers, clinical experts, frontline lecturers, and teaching administrators (all with ≥5 years of relevant experience). Interviews can be conducted in focus groups divided by population type or in mixed groups.

**Section 1: Ice-breaking and Basic Cognition**

1.How many years have you been involved in the access, use, development, or management of medical digital learning resources in your daily work? What are the main types of resources you have contact with (e.g., online courses, virtual simulation experiments, digital case libraries, micro-lectures, 3D anatomical resources, etc.)?

2.In your opinion, what are the core characteristics of a qualified and high-quality set of medical digital learning resources? What are the essential, discipline-specific differences between these resources and digital learning resources for non-medical disciplines?

**Probing Questions:**

Are there any unique requirements for medical disciplines that must be included in the quality evaluation system? What are the core pain points you have encountered when using or developing such resources?

**Section 2: Course Content Quality Dimension**

**Core** **Question**: For medical digital learning resources, what evaluation dimensions do you believe must be included at the course content level, and what specific evaluation points correspond to each dimension?

**Probing Questions:**

1.Medical knowledge has extremely high requirements for accuracy and rigor. In your opinion, what essential assessment points should be included regarding the scientific validity and completeness of the resource content?

2.Knowledge in the medical field is updated and iterated rapidly. How should the cutting-edge nature of resource content be evaluated, and what specific mandatory requirements should be set?

3.Beyond the knowledge itself, do you believe the resources need to reflect modern medical education concepts such as New Medical Science and information-based teaching? How should this aspect be evaluated?

4.How should the "student-centered" design be implemented and reflected in medical digital learning resources, in your opinion? What are the quantifiable and evaluable core points for this dimension?

5.The medical profession combines humanistic attributes and social responsibility. What evaluation elements should be included regarding the value orientation of the resources, such as the integration of curriculum ideological and political education, medical humanistic care, and responsiveness to primary healthcare and public health hotspots?

6.For the cultivation of medical students' ability to solve complex clinical problems and high-order clinical thinking, how should this be reflected in the resource content, and should it be included as a core evaluation dimension?

**Section 3: Curriculum and Teaching Design Dimension**

**Core Question**: At the curriculum and teaching design level, what core requirements must high-quality medical digital learning resources meet, and what evaluation dimensions must be included?

**Probing Questions:**

1.Which teaching methods (e.g., blended learning, flipped classroom, PBL/CBL/TBL, etc.) do you believe are more suitable for medical digital learning resources? How should the adaptability between teaching methods and medical teaching content and objectives be evaluated?

2.Medical education combines both theoretical and highly practical attributes. What evaluation points should be included regarding the diversity of resource forms, such as VR/3D anatomy, virtual simulation, micro-lectures, and other diversified formats?

3.For medical students of different majors, grades, and foundational levels, how should the personalized adaptation of resources and autonomous control of learning paths be evaluated?

4.Do you believe the measurability of learning outcomes should be included as a core evaluation dimension of teaching design, such as the supporting chapter question banks, video reviews of skill operations, and assessment system design?

**Section 4: Platform Technical Function Dimension**

**Core Question**: At the technical function level of the platform carrying digital learning resources, what core dimensions will directly affect the quality and application effect of the resources and must be included in the evaluation system?

**Probing Questions:**

1.Do you believe evaluation standards should be set for the operational convenience, navigation clarity, and operational smoothness of the platform? If yes, how should these standards be formulated?

2.Medical education has an extremely high demand for teacher-student and student-student collaboration. What essential evaluation points should be set for the platform's interactive functions and collaborative learning support (e.g., discussion forums, group task areas)?

3.Do you believe the visualized analysis of learning data (e.g., heat map of knowledge mastery, learning progress statistics, personal learning profiles) should be included in the evaluation? What are the core values of this function for medical education?

**Section 5: Artistry and Practicality Dimension**

**Core Question**: At the level of interface design, visual presentation, and technical practicality of the resources, what core points need to be included in the evaluation system?

**Probing Questions:**

1.Medical scenarios have their own professional particularities. Do you believe the aesthetic style, color matching, and information presentation method of the interface design should be included in the evaluation? If yes, what are the discipline-specific evaluation requirements?

2.For complex medical data, anatomical structures, epidemiological trends, and other content, should the visualized information presentation (e.g., standardized charts, 3D models) be included in the evaluation?

3.How should evaluation standards be set for the platform's response speed, fault resolution efficiency, and remote technical support services?

**Section 6: Resource Update and Feedback Dimension**

**Core Question**: At the level of dynamic resource update and teacher-student interactive feedback mechanisms, what core points must be included in the evaluation system?

**Probing Questions:**

1.Medical clinical practice guidelines, industry specifications, and research advances are updated frequently. How should the update frequency and update mechanism of resources be set and evaluated?

2.How should an effective teacher-student feedback mechanism be evaluated, such as the timeliness of teachers' answers to questions and whether a closed loop of "development-application-evaluation" is formed?

**Section 7: Population-Specific Targeted Probing (Selected Based on the Population of the Interview Group)**

**1.For Medical Education Researchers**: What core educational measurement principles do you believe this evaluation system should follow in indicator design to ensure its scientific validity and effectiveness? From the perspective of medical education reform trends, what other forward-looking dimensions need to be included?

**2.For Clinical Experts**: From the perspective of clinical post competency cultivation, what indicators aligned with clinical practice should be the focus of quality evaluation for digital learning resources? What are the core evaluation points for digital resources related to the cultivation of clinical skills and diagnostic thinking?

**3.For Frontline Lecturers**: From the perspective of classroom teaching implementation, what essential evaluation points should be set for the classroom usability and teaching adaptability of digital learning resources? What are the characteristics you value most and the defects you find most unacceptable when using digital resources in teaching?

**4.For Teaching Administrators**: From the perspective of institutional teaching resource management, promotion and application, what dimensions should be included in the evaluation system? What essential evaluation points must be set for the copyright specifications, data security, and institutional-wide promotion adaptability of the resources?

**Section 8: Supplement and Closing**

1.In addition to the dimensions we discussed above, what other core evaluation dimensions or indicators do you believe must be included in this quality evaluation system for medical digital learning resources?

2.Do you have any other suggestions, supplements, or reminders for the construction of this evaluation system?

Thank you very much for your valuable sharing. Your perspectives are critical to our research. We may invite you to participate again in the subsequent Delphi expert consultation of this study. Thank you again for your support!

**Outline 2: Interview Outline for Medical Students**

**Target Population**: Enrolled medical students

**Section 1: Ice-breaking and Basic Cognition**

1.How long have you been using medical digital learning resources? What types of resources do you use most frequently in your daily study (e.g., online courses, virtual simulation experiments, digital anatomy libraries, micro-lectures, clinical case libraries, question banks, etc.)? What is your approximate frequency of use?

Based on your user experience, what are the core differences between "high-quality, user-friendly" medical digital learning resources and "low-quality, poor-performing" ones?

**2.Section 2: Content Quality Dimension**

**Core Question**: In your opinion, what aspects do you value most when judging the quality of the content of a medical digital teaching resource?

**Probing Questions**:

1.How important is the accuracy of the resource content and the completeness of knowledge points to you? Have you ever encountered errors or outdated content in the resources you used?

2.Does the inclusion of the latest medical advances and clinical practice guidelines in the resources have a significant impact on your study?

3.Does the resource integrate content related to clinical cases, medical humanistic care, and professional literacy? Do you think this integration is necessary? Is it helpful for your study?

4.Is the resource designed with a student-centered approach, for example, broken down into micro-lectures and case analyses, to help you better understand knowledge and develop clinical thinking?

5.Does the resource cover content related to primary healthcare skills and public health hotspots? Do you think this should be included?

**Section 3: Teaching Design and Learning Support Dimension**

**Core Question**: During your study, what aspects of the resource's teaching design and learning support will directly affect your learning outcomes and learning willingness?

**Probing Questions:**

1.Can the teaching methods used in the resources, such as case-based learning, scenario simulation, and flipped classroom, help you better grasp knowledge? Which learning format do you prefer?

2.Are the resource forms diverse, such as 3D anatomy, virtual simulation, videos, images and texts? How much do these help your study?

3.Are the resources divided by difficulty levels, and can you arrange your own learning progress and plan a personalized learning path that suits you?

4.Are there supporting chapter tests, skill operation reviews, and assessment content to help you test your learning outcomes? Do you think this function is important?

**Section 4: Platform Technology and User Experience Dimension**

**Core Question**: What aspects of the platform and interface carrying the resources will directly affect your user experience and learning efficiency? Do you think these functions are important?

**Probing Questions:**

1.Is the platform easy to operate, with clear navigation and smooth operation? Does it often freeze or fail to open?

Is the interface design user-friendly with clear information presentation? Does it convert complex medical knowledge into charts and visualized content to help you understand better?

2.Does the platform have interactive functions, such as discussion forums and group task areas, to facilitate interactive communication and collaborative learning with teachers and classmates?

3.Is there statistical analysis of learning data and visualized presentation of knowledge mastery to help you understand your learning progress and weak points?

**Section 5: Resource Update and Feedback Dimension**

**Core Question**: During your study, do you pay attention to the resource updates and teachers' feedback? What are your core requirements for this aspect?

**Probing Questions:**

1. Will the outdated content of resources, without updates to the latest medical knowledge and guidelines, affect your use? How often do you think resources should be updated? Do you think the feedback time should be included as an indicator for evaluating the quality of digital learning resources?

2. After you ask teachers questions during your study, does the timeliness of their replies have a great impact on your study? What do you think is a reasonable response time frame?

**Section 6: Supplement and Closing**

1. In addition to the content we discussed above, what other aspects do you think must be considered when evaluating the quality of medical digital learning resources?
2. Do you have any other critical feedback, suggestions, or expectations for medical digital learning resources?

Thank you very much for your valuable sharing. Your real user experience is extremely important to our research, and we will incorporate your suggestions into the construction of the evaluation system.

Thank you again for your participation!
