# Supplementary material for: Development and validation of a digital learning resource quality evaluation framework: a mixed-methods study in health professions education
Source: Front Public Health. 2026 Oct 14;14:1958724. doi: 10.3389/fpubh.2026.1958724 (PMC13715655; doi:10.3389/fpubh.2026.1958724)
Supplement: Supplementary file 8 [file Table_3.DOCX]

**Supplementary material 3: Demographic characteristics of participants**

Table S3.1 Demographic characteristics of focus group interview experts

| Category | Stakeholder Role | Number | Experience (years, $\overline{X}\pm S$) |
| --- | --- | --- | --- |
| Experts | Medical education researchers | 4 | 18.5±6.2 |
| Experts | Clinical medicine experts | 4 | 15.3±5.8 |
| Experts | Frontline teaching experts | 4 | 12.7± 4.3 |
| Experts | Education administrators | 3 | 14.0± 5.1 |
| Total |  | 15 |  |

Table S3.2 Demographic characteristics of focus group interview students

| Major | Freshman | Sophomore | Junior | Senior | Total |
| --- | --- | --- | --- | --- | --- |
| Clinical Medicine | 5 | 4 | 6 | 8 | 23 |
| Preventive Medicine | 9 | 8 | 0 | 10 | 27 |
| Total | 14 | 12 | 6 | 18 | 50 |

Table S3.3 Distribution of students by major and academic year (n = 309)

| Major | Sophomore | Junior | Senior | Total |
| --- | --- | --- | --- | --- |
| Clinical Medicine | 80 | 76 | 0 | 156 |
| Preventive Medicine | 0 | 0 | 153 | 153 |
| **Total** | **80** | **76** | **153** | **309** |

Note that academic year and program were partially confounded by design: clinical-medicine students were in Years 2–3, whereas preventive-medicine students were in Year 4, reflecting the curricular sequencing of the two programs; between-program comparisons should therefore be interpreted with this caveat.
