# Supplementary material for: Development and validation of a digital learning resource quality evaluation framework: a mixed-methods study in health professions education
Source: Front Public Health. 2026 Oct 14;14:1958724. doi: 10.3389/fpubh.2026.1958724 (PMC13715655; doi:10.3389/fpubh.2026.1958724)
Supplement: Supplementary file 9 [file Table_4.DOCX]

**Supplementary Material 4: Delphi Questionnaire and Validation scale**

**4.1Round 1 Delphi Questionnaire**

**Invitation for Expert Consultation:
Development of a Quality Evaluation System for Digital Teaching Resources in Medical Institutions (First Round)**

Distinguished Expert:

This study aims to evaluate the quality of medical digital learning resources. Your participation is entirely voluntary. All responses are anonymous, and no identifiable personal data will be collected. The data will be used exclusively for academic research purposes. By proceeding to complete and submit this questionnaire, you indicate that you have read and understood this information and consent to participate. If you do not wish to participate, please do not submit the questionnaire.

This study has been approved by the Medical Ethics Committee of Kunming Medical University (Approval Number: KMU2026MEC001).

Thank you for your support!

**Instructions:**

The index system is divided into three levels (5 first-level indexes, 11 second-level indexes, 21 third-level indexes).

Importance score: 5= very important, 4= relatively important, 3= general, 2= relatively unimportant, 1= unimportant.

To modify or add indicators, please enter specific suggestions in the corresponding columns and score them.

**Expert Demographic Information**

| Professional Title |  | Age |  |
| --- | --- | --- | --- |
| Work Position | A: Administration B: Teaching & Research C: Professional Technical D: Other | | |
| Research Field |  | | |
| Years of Experience in This Position |  | | |

Note: Mark the appropriate impact level with a "√" based on the criteria. Only one "√" can be marked per criterion.

**Self-assessment of Expert Judgment Basis**

| Judgment Basis | Degree of Influence | | |
| --- | --- | --- | --- |
|  | High | Medium | Low |
| Work Experience |  |  |  |
| Theoretical Analysis |  |  |  |
| Domestic & International References |  |  |  |
| Intuitive Feeling |  |  |  |

**Self-assessment of Expert Familiarity**

| Familiarity Level | Very Familiar | Somewhat Familiar | Neutral | Somewhat Unfamiliar | Very Unfamiliar |
| --- | --- | --- | --- | --- | --- |
| Expert Self-evaluation |  |  |  |  |  |

| **First-level indicator** | **Indicator connotation** | **Indicator importance (decreasing order)** | | | | | **suggestions on revision** | |
| --- | --- | --- | --- | --- | --- | --- | --- | --- |
|  |  | 5 | 4 | 3 | 2 | 1 | **revise content** | **grade** |
| 1. Quality of the course content | Evaluate the scientific nature and educational value of teaching resources in terms of the medical knowledge system, educational concepts, and value orientation. |  |  |  |  |  |  |  |
| 2. Course/Teaching Design | Evaluate the applicability of teaching methods, the diversity of resources, and the degree of support for learners' individualized needs. |  |  |  |  |  |  |  |
| 3.Platform Technical Functions | Assess the technical stability of the platform, its interactive features, and data support capabilities |  |  |  |  |  |  |  |
| 4. Artistry and Practicality | Evaluate the visual effects and functional practicality of the interface design. |  |  |  |  |  |  |  |
| 5. Resource updates and feedback | Assess the mechanism for resource updates and the efficiency of interaction and feedback between teachers and students. |  |  |  |  |  |  |  |
| Other suggestions: | | | | | | | | |
| **Second-level indicator** | **Indicator connotation** | **Indicator importance (decreasing order)** | | | | | **suggestions on revision** | |
|  |  | 5 | 4 | 3 | 2 | 1 | **revise content** | **grade** |
| 1.1 Educational Philosophy | Does the resource reflect modern medical education concepts (such as new medical science, information-based teaching) and a student-centered design approach? |  |  |  |  |  |  |  |
| 1.2 Value Orientation | Does the resource incorporate ideological and political education, medical humanistic care, and respond to social demands (such as primary healthcare, public health hotspots)? |  |  |  |  |  |  |  |
| 1.3 Content Design | Accuracy, logic, and cutting-edge nature of the content, and whether it conforms to the characteristics of medical disciplines and learning cognitive laws. |  |  |  |  |  |  |  |
| Other suggestions: |  | | | | | | | |
| 2.1 Teaching Methods | Does it adopt advanced teaching methods (such as blended learning) and are they highly compatible with medical teaching content? |  |  |  |  |  |  |  |
| 2.2 Resource Compatibility | Are the resource forms diverse (such as VR, micro-lectures), and do they support learners' autonomous control over their learning paths? |  |  |  |  |  |  |  |
| Other suggestions: |  | | | | | | | |
| 3.1 Interactive Experience | Is the platform user-friendly and smooth, and does it provide learning data visualization analysis functions? |  |  |  |  |  |  |  |
| 3.2 Collaboration Support | Does it support collaborative learning among teachers and students/students and students (such as discussion forums, group tasks)? |  |  |  |  |  |  |  |
| Other suggestions: | not have | | | | | | | |
| 4.1 Interface Design | Does the interface conform to the aesthetic standards of medical scenarios, and is the information presentation clear and intuitive (such as charted data)? |  |  |  |  |  |  |  |
| 4.2 Technical Support | Platform response speed, fault resolution efficiency, and the convenience of remote assistance services. |  |  |  |  |  |  |  |
| Other suggestions: | not have | | | | | | | |
| 5.1 Resource Updates | Are core resources updated regularly, and do they keep pace with medical advancements and changes in teaching demands? |  |  |  |  |  |  |  |
| 5.2 Feedback Mechanism | Do teachers respond to students' questions in a timely manner, and is there an effective "build-use-evaluate" closed loop formed? |  |  |  |  |  |  |  |
| Other suggestions: |  | | | | | | | |
| **Third-Level indicator** | **Indicator connotation** | **Indicator importance (decreasing order)** | | | | | **suggestions on revision** | |
|  |  | 5 | 4 | 3 | 2 | 1 | **revise content** | **grade** |
| 1.1.1 Advancement | It reflects the new medical education and information-based teaching concepts. |  |  |  |  |  |  |  |
| 1.1.2 Student-centered | Student-centered design (e.g. micro-videos, case studies). |  |  |  |  |  |  |  |
| 1.2.1 Integration of Ideological-Political Education | Illustrate professional ethics, medical equity, and the Healthy strategy through cases. |  |  |  |  |  |  |  |
| 1.2.2 Responsiveness to Societal Needs | Cover primary medical skills and public health hotspots. |  |  |  |  |  |  |  |
| 1.3.1 Scientificity | The knowledge points are complete and accurate, and the logic is clear. |  |  |  |  |  |  |  |
| 1.3.2 Frontier | International medical advance and the latest research results. |  |  |  |  |  |  |  |
| 1.3.3 High-order | Cultivate the comprehensive ability and advanced thinking ability to solve complex problems. |  |  |  |  |  |  |  |
| Other suggestions: |  | | | | | | | |
| 2.1.1 Methodological Advancement | Adopting digital teaching methods such as flipped classroom and blended learning, it supports multiple learning paths. |  |  |  |  |  |  |  |
| 2.1.2 Alignment with Curriculum | The teaching method is highly matched with the teaching content. |  |  |  |  |  |  |  |
| 2.2.1 Resource Diversity | Include diverse resources such as micro-lectures, videos/3D anatomy, etc. |  |  |  |  |  |  |  |
| 2.2.2 Resource Adaptability | Differentiate professional difficulty levels, and mark suggested learning durations and prerequisite knowledge. |  |  |  |  |  |  |  |
| 2.2.3 Learner Control | Personalized learning paths |  |  |  |  |  |  |  |
| 2.2.4 Measurability of Learning Outcomes | Equipped with chapter test question banks, skill operation video replays and comments, etc. |  |  |  |  |  |  |  |
| Other suggestions: |  | | | | | | | |
| 3.1.1 Accessibility | Clear navigation and smooth operation. |  |  |  |  |  |  |  |
| 3.1.2 Level of Interactive Engagement | High proportion of student-teacher interaction in the classroom. |  |  |  |  |  |  |  |
| 3.1.3 Data Visualization | Generate personal learning profiles (e.g., knowledge mastery heatmaps) and progress statistics. |  |  |  |  |  |  |  |
| 3.2.1 Collaborative Learning Function | Support platform communication functions (discussion area, group task area). |  |  |  |  |  |  |  |
| Other suggestions: |  | | | | | | | |
| 4.1.1 Interface Aesthetics | Main color tones consistent with medical scenarios (such as operating room gray/blue). |  |  |  |  |  |  |  |
| 4.1.2 Information Visualization | Convert complex data into charts (e.g. epidemiological trend line charts). |  |  |  |  |  |  |  |
| Other suggestions: |  | | | | | | | |
| 5.1.1 Update Frequency | Core resources update with each teaching cycle, and teaching materials are updated in real time. |  |  |  |  |  |  |  |
| 5.2.1 Timeliness of Teacher Response | Efficiency of teachers' responses to discussion questions. |  |  |  |  |  |  |  |
| Other suggestions: |  | | | | | | | |

**Supplementary Material 4:**

**4.2 Round 2 Delphi Questionnaire**

**Invitation for Expert Consultation:
Development of a Quality Evaluation System for Digital Teaching Resources in Medical Institutions (Second Round)**

Distinguished Expert:

This study aims to evaluate the quality of medical digital learning resources. Your participation is entirely voluntary. All responses are anonymous, and no identifiable personal data will be collected. The data will be used exclusively for academic research purposes. By proceeding to complete and submit this questionnaire, you indicate that you have read and understood this information and consent to participate. If you do not wish to participate, please do not submit the questionnaire.

This study has been approved by the Medical Ethics Committee of Kunming Medical University (Approval Number: KMU2026MEC001).

Thank you for your support!

**Instructions:**

The index system is divided into three levels (4 first-level indexes, 10 second-level indexes, 14 third-level indexes).

Importance score: 5= very important, 4= relatively important, 3= general, 2= relatively unimportant, 1= unimportant.

To modify or add indicators, please enter specific suggestions in the corresponding columns and score them.

**Expert Demographic Information**

| Professional Title |  | Age |  |
| --- | --- | --- | --- |
| Work Position | A: Administration B: Teaching & Research C: Professional Technical D: Other | | |
| Research Field |  | | |
| Years of Experience in This Position |  | | |

Note: Mark the appropriate impact level with a "√" based on the criteria. Only one "√" can be marked per criterion.

**Self-assessment of Expert Judgment Basis**

| Judgment Basis | Degree of Influence | | |
| --- | --- | --- | --- |
|  | High | Medium | Low |
| Work Experience |  |  |  |
| Theoretical Analysis |  |  |  |
| Domestic & International References |  |  |  |
| Intuitive Feeling |  |  |  |

**Self-assessment of Expert Familiarity**

| Familiarity Level | Very Familiar | Somewhat Familiar | Neutral | Somewhat Unfamiliar | Very Unfamiliar |
| --- | --- | --- | --- | --- | --- |
| Expert Self-evaluation |  |  |  |  |  |

| **First-level**  **indicator** | **Second-level indicator** | **Third-Level indicator** | **Indicator connotation** | **Indicator importance (decreasing order)** | | | | | **suggestions on revision** | |
| --- | --- | --- | --- | --- | --- | --- | --- | --- | --- | --- |
|  |  |  |  | 5 | 4 | 3 | 2 | 1 | **revise content** | **grade** |
| 1. Content quality | 1.1 Educational Philosophy | 1.1.1 Advancedness | It reflects the new medical education and information-based teaching concepts. |  |  |  |  |  |  |  |
|  |  | 1.1.2 Student-centered | Centered on students' learning and development, the design of case studies and micro-lectures should support the cultivation of job competencies. |  |  |  |  |  |  |  |
|  | 1.2 Content Scienticicity | 1.2.1 Scientific Accuracy | Integrate professional hotspots and incorporate professional ethics, the spirit of scientists, medical humanistic care, and the Healthy strategy, etc. |  |  |  |  |  |  |  |
|  |  | 1.2.2 Updated Content & Frontier Knowledge | Cover primary medical skills and public health hotspots. |  |  |  |  |  |  |  |
|  | 1.3 Professional Ethics & Values | 1.3.1 Professional Ethics & Values Education | The knowledge points should be complete and accurate, meeting the needs of professional talent cultivation. |  |  |  |  |  |  |  |
|  |  | 1.3.2 Social Responsiveness | International medical progress and the latest research results. |  |  |  |  |  |  |  |
| 2. Instructional Design | 2.1 Teaching Methods | 2.1.1 Methodological Appropriateness | The alignment of blended learning, flipped classrooms, PBL/CBL/TBL, group discussions, brainstorming, scenario simulations, and inquiry-based teaching with course content and instructional objectives demonstrates their higher-order cognitive engagement. |  |  |  |  |  |  |  |
|  | 2.2 Resource Diversity | 2.2.1 Resource Formats | Featuring cutting-edge resources including VR, 3D anatomy, virtual simulations, MOOCs, interdisciplinary case studies, and AI-powered innovations. |  |  |  |  |  |  |  |
|  | 2.3 Learning Support | 2.3.1 Personalized Learning | The assignment difficulty classification, self-controlled learning path and progress, and learning achievement evaluation (test, design plan, etc.) can promote students' active learning. |  |  |  |  |  |  |  |
|  |  | 2.3.2Interaction Design |  |  |  |  |  |  |  |  |
| 3. Evaluation and Feedback | 3.1 Learning Evaluation | 3.1.1 Multiple Evaluations | Adopt a combination of formative assessment (such as chapter quizzes, case analysis reports, etc.) and summative assessment (final exams). |  |  |  |  |  |  |  |
|  | 3.2 Application Feedback | 3.2.1 Timeliness & Update Adaptability | Core resources are updated regularly, and teaching materials are adjusted in real time. The extent of teaching resource application before, during, and after class. |  |  |  |  |  |  |  |
| 4. Platform Features | 4.1 Interactive Experience | 4.1.1 Compatibility & Security | Clear navigation, convenient operation, and privacy protection (student information, teachers' original resource copyrights, etc.). |  |  |  |  |  |  |  |
|  |  | 4.1.2 Data Visualization | Visualize learning profiles and progress statistics, and provide real-time feedback after answering questions. |  |  |  |  |  |  |  |

**Supplementary Material 4**

**4.3 Validation scale**

**Invitation for Validation scale**

Dear expert/student:

To scientifically evaluate the quality of digital teaching resources and enhance teaching effectiveness, we have developed this evaluation system. Your participation is entirely voluntary. All responses are anonymous, and no identifiable personal data will be collected. The data will be used exclusively for academic research purposes. By proceeding to complete and submit this scale, you indicate that you have read and understood this information and consent to participate. If you do not wish to participate, please do not submit the questionnaire.

This study has been approved by the Medical Ethics Committee of Kunming Medical University (Approval Number: KMU2026MEC001).

Thank you for your support!

filling explanation：

1. Please score for only one course's digital teaching resources. If you are familiar with multiple courses, prioritize the one with the longest usage duration.

2. All questions are mandatory. There is no right or wrong answer—just fill in based on your actual feelings.

3. The response time should be approximately 2-5 minutes. If you leave the questionnaire midway, it will be considered invalid.

System description:

Evaluation object: Digital teaching resources you are familiar with or have studied (multiple selections allowed: □ Online courses □ Virtual simulation experiments □ Digital case libraries □ Others: )

**Course Title：**	(Required, such as Internal Medicine, Epidemiology)

Evaluator identity: □ student □ expert (medical education/clinical or preventive medicine teacher/teaching management)

**If you are a student:**

Year: □ sophomore □ junior □ senior □ Other: ______

Major: □ Clinical Medicine □ Preventive Medicine

Duration of resource use: □1-4 weeks □5-8 weeks □9-12 weeks □12 weeks or more

**If you are an expert,**

Professional fields: □ Medical education research □ Clinical medicine □ Preventive medicine □ Teaching management □ Others: ______

Years of service: □Less than 5 years □5-10 years □More than 10 years

Scoring criteria: A 5-point Likert scale is used, with the following specific definitions:

1= Completely non-compliant: The resource fails to meet the specified requirements and has serious defects;

2=Not fully compliant: The resource section meets the requirements, but the core functions are missing or significantly inadequate;

3=General: Resources meet basic requirements with no major defects but significant room for optimization;

4=Fairly meets the requirements: The resources are adequate, core functions are complete, with only minor details needing refinement.

5= Fully compliant: Resources fully meet or exceed requirements, with excellent quality.

Please rate the resource objectively based on its actual condition.

| **First-level**  **indicator** | **Second-level indicator** | **Third-Level indicator** | **Indicator Connotation (operational definition)** | **grade**  **(1-5 points)** |
| --- | --- | --- | --- | --- |
| 1. Content quality | 1.1 Educational Philosophy | 1.1.1 Advancedness | The integration of new medical education concepts with digital and information-based teaching technologies demonstrates the innovation and upgrading of traditional teaching methods, aligning with the reform needs of medical talent cultivation models. |  |
|  |  | 1.1.2 Student-centered | Centered on students' learning outcomes and vocational intelligence development, this approach utilizes digital platforms to precisely align with core competency requirements, fostering personalized growth and professional competence cultivation. |  |
|  | 1.2 Scientific Content | 1.2.1 Scientificity | The core knowledge points are presented comprehensively and accurately (free from academic disputes and factual errors), aligning with medical education programs, curriculum standards, and professional practice guidelines, thereby supporting the systematic construction of the knowledge framework. |  |
|  |  | 1.2.2 Frontier | It includes the international and domestic medical advances, academic research achievements and professional technical innovations in the past three years, ensuring that the content of resources keeps pace with the development of medicine. |  |
|  | 1.3 Ideological and Political Education and Humanities | 1.3.1 Ideological and Political Guidance | Closely aligned with the hotspots of medical professions and professional demands, it organically integrates professional spirit, scientific spirit, medical humanistic care, and the strategic concept of health China, achieving the alignment of professional knowledge transmission and value guidance. |  |
|  |  | 1.3.2 Social Response | Focus on the core skills of primary health care services, accurately respond to health-related social hot issues, and improve the social service value and practical adaptability of resources. |  |
| 2. Instructional Design | 2.1 Teaching Methods | 2.1.1 Methodological Support | The resource design is fully adapted to the teaching process, directly supporting the implementation of PBL/CBL and other teaching methods. It provides modular medical case materials, interactive discussion task sheets and other supporting materials with high integration and ready-to-use features. |  |
|  | 2.2 Resource Diversity | 2.2.1 Forms of Resources | The resources are rich and diverse, which can support the cultivation of higher cognitive abilities (such as decision-making ability and critical thinking). |  |
|  | 2.3 Learning Support | 2.3.1 Personalization | ① Resources are organized by difficulty levels: basic, intermediate, and advanced; ② Assessments (e.g., question banks, assignments) are modular, allowing teachers to design personalized learning plans. |  |
|  |  | 2.3.2 Interaction | The resources include interactive materials such as discussion, collaboration, and feedback, which can directly support scenarios like teacher-student discussions and peer collaboration. |  |
| 3. Evaluation and Feedback | 3.1 Learning Evaluation | 3.1.1 Multiple Evaluations | The resources include supporting materials for multiple evaluations, which can support the implementation of formative and summative evaluations, demonstrating the completeness and adaptability of the evaluation materials. |  |
|  | 3.2 Application Feedback | 3.2.1 Update and Application | 1.Timeliness: Includes frontier medical achievements and policy updates (e.g., new guidelines) from the past three years, with no outdated information;  2.Update adaptability: The resource format supports modular updates, eliminating the need for complete reconstruction and reducing update costs. |  |
| 4. Platform Features | 4.1 Interactive Experience | 4.1.1 User-friendly interface | 1.The resource format is compatible with mainstream digital teaching platforms, with no opening or playback issues;  2. The resource includes a quick location feature for efficient use;  3.It contains copyright notices and student personal information usage guidelines, meeting cybersecurity and privacy protection requirements. |  |
|  |  | 4.1.2 Data Visualization | The resource includes a learning feedback module, which provides core data support for visualizing learning progress and enabling personalized adjustments, demonstrating the 'intelligent feedback' advantage of digital resources. |  |

Suggestions

1. Which indicator in this evaluation system do you think best reflects the quality of digital teaching resources? Why?

________________________________________________________________

2. What other important indicators do you think are missing from this evaluation system? Please provide additional information.

________________________________________________________________

3. Other recommendations:

________________________________________________________________

________________________________________________________________
