# Supplementary material for: Development and validation of a digital learning resource quality evaluation framework: a mixed-methods study in health professions education
Source: Front Public Health. 2026 Oct 14;14:1958724. doi: 10.3389/fpubh.2026.1958724 (PMC13715655; doi:10.3389/fpubh.2026.1958724)
Supplement: Supplementary file 10 [file Table_5.DOCX]

Supplementary material 5:**Calculation Steps of the Analytic Hierarchy Process (AHP)**

The Analytic Hierarchy Process (AHP), originally developed by Saaty, was applied to derive the subjective (expert) weights of the indicator system. Based on the Round 2 Delphi survey, 15 experts performed pairwise comparisons among criteria within each judgment matrix using Saaty’s 1-9 fundamental scale (1 = equal importance, 9 = absolute importance). Individual expert judgments were aggregated into a group judgment matrix using the geometric mean. Local weights were then obtained by the square-root (geometric mean) method, and the consistency of each judgment matrix was assessed using the consistency ratio (CR).

**Step 1: Construction of the pairwise comparison (judgment) matrix**

For a set of n criteria compared at the same decision node, the pairwise comparison matrix is defined as

$$\mathbf{A}=\left[ a_{ij} \right]_{n\times n}=\left[ \begin{matrix} 1 & a_{12} & \cdots& a_{1n} \\ a_{21} & 1 & \cdots& a_{2n} \\ \vdots& \vdots& \ddots& \vdots\\ a_{n1} & a_{n2} & \cdots& 1 \end{matrix} \right].$$

Here, $a_{\mathrm{ij}}$ denotes the relative importance of criterion $i$ with respect to criterion $j$. The judgment matrix satisfies the reciprocal conditions

$$a_{ji}=\frac{1}{a_{ij}}, a_{ii}=1.$$

Table S5-1. Saaty’s 1-9 fundamental scale used for pairwise comparisons.

| **Intensity of importance** | **Definition** | **Explanation** |
| --- | --- | --- |
| 1 | Equal importance | Two criteria contribute equally to the objective. |
| 3 | Moderate importance | Experience and judgment slightly favor one criterion over another. |
| 5 | Strong importance | Experience and judgment strongly favor one criterion over another. |
| 7 | Very strong importance | One criterion is strongly favored and its dominance is demonstrated in practice. |
| 9 | Absolute importance | The highest possible order of affirmation of one criterion over another. |
| 2, 4, 6, 8 | Intermediate values | Compromise values between two adjacent judgments. |

**Step 2: Aggregation of expert judgments**

Let $a_{\mathrm{ij}}^{\left( k \right)}$ denote the judgment of expert $k$ for the pair $\left( i,j \right)$. The 15 individual judgments for each matrix element were aggregated using the geometric mean:

$$\bar{a}_{ij}=\left( \prod_{k=1}^{m} a_{ij}^{\left( k \right)} \right)^{1/m}, m=26.$$

The resulting group judgment matrix is

$$\bar{\mathbf{A}}=\left[ \bar{a}_{ij} \right]_{n\times n}.$$

The geometric-mean aggregation preserves reciprocity because

$$\bar{a}_{ji}=\frac{1}{\bar{a}_{ij}}.$$

**Step 3: Calculation of local weights by the square-root (geometric mean) method**

For each criterion $i$, the unnormalized weight is calculated as the geometric mean of the entries in row $i$ of the aggregated judgment matrix:

$$\tilde{w}_{i}=\left( \prod_{j=1}^{n} \bar{a}_{ij} \right)^{1/n}.$$

The normalized local AHP weight is then obtained as

$$w_{i}=\frac{\tilde{w}_{i}}{\sum_{r=1}^{n} \tilde{w}_{r}}, \sum_{i=1}^{n} w_{i}=1.$$

Here, $n$ is the order of the judgment matrix currently being evaluated. For example, $n=4$ for the first-level judgment matrix. For lower-level matrices in a hierarchical AHP model, $n$ equals the number of sibling criteria compared under the same parent criterion.

**Step 4: Calculation of the maximum eigenvalue (**$\boldsymbol{\lambda}_{\mathbf{max}}$**)**

Let

$$\mathbf{W}=\left( w_{1},w_{2},\ldots,w_{n} \right)^{T}.$$

First compute

$$\bar{\mathbf{A}}\mathbf{W}=\left[ \begin{matrix} \left( \bar{\mathbf{A}}\mathbf{W} \right)_{1} \\ \left( \bar{\mathbf{A}}\mathbf{W} \right)_{2} \\ \vdots\\ \left( \bar{\mathbf{A}}\mathbf{W} \right)_{n} \end{matrix} \right].$$

The maximum eigenvalue is estimated as

$$\lambda_{\max}=\frac{1}{n}\sum_{i=1}^{n} \frac{\left( \bar{\mathbf{A}}\mathbf{W} \right)_{i}}{w_{i}}.$$

**Step 5: Consistency test**

The consistency index (CI) is calculated as

$$CI=\frac{\lambda_{\max}-n}{n-1}.$$

The consistency ratio (CR) is calculated as

$$CR=\frac{\mathrm{CI}}{\mathrm{RI}},$$

where RI is the random index corresponding to matrix order $n$. A judgment matrix is considered acceptably consistent when

$$CR<0.10.$$

If $CR\geq0.10$, the pairwise comparisons should be reviewed and revised before the weights are accepted.

Table S5-2. Random index (RI) values used for the consistency test.

| **n (matrix order)** | **RI** |
| --- | --- |
| 1 | 0.00 |
| 2 | 0.00 |
| 3 | 0.58 |
| 4 | 0.90 |
| 5 | 1.12 |
| 6 | 1.24 |
| 7 | 1.32 |
| 8 | 1.41 |
| 9 | 1.45 |
| 10 | 1.49 |
| 11 | 1.52 |
| 12 | 1.54 |
| 13 | 1.56 |
| 14 | 1.59 |

**Synthesis of global weights in the multi-level hierarchy**

When lower-level indicators are evaluated through separate local judgment matrices, their global AHP weights are obtained by multiplying the local weight of the indicator by the local weights of all ancestor criteria along its hierarchy path. For a path containing $L$ hierarchical levels,

$$w_{i}^{\left( \mathrm{global} \right)}=\prod_{\ell=1}^{L} w_{i,\ell}^{\left( \mathrm{local} \right)}.$$

The resulting global weights may then be normalized across the set of terminal indicators when a common comparison scale is required.
