# Supplementary material for: Development and validation of a digital learning resource quality evaluation framework: a mixed-methods study in health professions education
Source: Front Public Health. 2026 Oct 14;14:1958724. doi: 10.3389/fpubh.2026.1958724 (PMC13715655; doi:10.3389/fpubh.2026.1958724)
Supplement: Supplementary file 11 [file Table_6.DOCX]

**Supplementary material 6: Calculation Steps of the Entropy Weight Method (EWM)**

The calculation was based on the Round 2 raw importance score matrix from the 26 Delphi experts, where each expert rated each indicator on a 5-point Likert scale (1 = completely unnecessary to 5 = highly necessary).

Step 1: Data Standardization

Since all indicators were positive, the raw scores were normalized using the Min-Max method to eliminate dimensional effects and ensure comparability across indicators:

where:

*X_ij_* is the raw score assigned by the *i*-th expert to the *j*-th indicator;

min(*X_j_*) and max(*X_j_*) are the minimum and maximum scores for the *j*-th indicator across all experts;

*P_ij_* is the normalized value, ranging from 0 to 1.

Step 2: Calculation of the Proportion Matrix

For each indicator, the normalized score of each expert was divided by the sum of all experts‘ normalized scores for that indicator, yielding the proportion matrix:

where:

P*ij* is the proportion of the ii-th expert‘s normalized score relative to the total for the *j*-th indicator, satisfying $\sum_{i=1}^{m}$P*_ij_*=1;

*m* is the total number of experts (in this study, *m*=26).

Step 3: Calculation of Information Entropy

The information entropy of each indicator was calculated to quantify the degree of dispersion (uncertainty) in expert responses:

$$e_{j}=-k\sum_{i=1}^{n} p_{ij}\ln(p_{ij})\text{，where }k=\frac{1}{\ln n}$$

where:

*e_j_* is the entropy value of the *j*-th indicator, ranging from 0 to 1;

*ln* denotes the natural logarithm;

*k* is the entropy normalization coefficient, ensuring that *e_j_*$\in$[0,1]

Interpretation: A higher entropy value (*e_j_* close to 1) indicates low dispersion among expert ratings (high consensus), meaning the indicator provides limited discriminatory information. Conversely, a lower entropy value (*e_j_* close to 0) indicates high dispersion (low consensus), meaning the indicator provides substantial discriminatory information.

Step 4: Calculation of the Dispersion Coefficient

The entropy value was transformed into a dispersion coefficient (also called the information utility value):

where *g_j_* represents the degree of differentiation of the jj-th indicator. A larger *g_j_* indicates greater discriminatory power and thus greater importance.

Step 5: Calculation of the EWM Objective Weight

Finally, the EWM objective weight for each indicator was obtained by normalizing the dispersion coefficients:

$$W_{\text{EWM}}=\frac{1-e_{j}}{\sum_{j=1}^{m} 1-e_{j}}$$

where:

w*_EWM_* is the objective weight of the *j*-th indicator derived from EWM;

*n* is the total number of indicators (in this study, *n*=14 tertiary indicators);

the denominator ensures that all weights sum to 1, i.e., $\sum_{j=1}^{n}$w*_EWM_* =1
