# Supplementary material for: Development and validation of a digital learning resource quality evaluation framework: a mixed-methods study in health professions education
Source: Front Public Health. 2026 Oct 14;14:1958724. doi: 10.3389/fpubh.2026.1958724 (PMC13715655; doi:10.3389/fpubh.2026.1958724)
Supplement: Supplementary file 12 [file Table_7.DOCX]

**Supplementary Material 7**

# **7.1 Expert consistency (CR) test**

Table S7.1 Consistency ratios (CR) of individual expert judgment matrices (n = 26).

| **Expert** | **CR** | **Result** |
| --- | --- | --- |
| E01 | 0.043 | Pass |
| E02 | 0.072 | Pass |
| E03 | 0.002 | Pass |
| E04 | 0.005 | Pass |
| E05 | 0.005 | Pass |
| E06 | 0.144 | Exclude |
| E07 | 0.072 | Pass |
| E08 | 0.203 | Exclude |
| E09 | 0.114 | Exclude |
| E10 | 0.022 | Pass |
| E11 | 0.112 | Exclude |
| E12 | 0.128 | Exclude |
| E13 | 0.150 | Exclude |
| E14 | 0.043 | Pass |
| E15 | 0.022 | Pass |
| E16 | 0.072 | Pass |
| E17 | 0.073 | Pass |
| E18 | 0.219 | Exclude |
| E19 | 0.070 | Pass |
| E20 | 0.000 | Pass |
| E21 | 0.115 | Exclude |
| E22 | 0.144 | Exclude |
| E23 | 0.043 | Pass |
| E24 | 0.183 | Exclude |
| E25 | 0.152 | Exclude |
| E26 | 0.043 | Pass |

Note. CR = CI/RI, where CI = (λ_max_ − 4) / 3 and RI = 0.90 for a 4×4 pairwise comparison matrix; λ_max_ denotes the maximum eigenvalue. CR < 0.10 indicates acceptable consistency. 11 experts had CR ≥ 0.10 and were excluded from the consistent subset; the remaining 15 experts formed the consistent subset used in subsequent analyses. Pass = retained in the consistent subset; Excluded = removed on consistency grounds.

# **7.2 Aggregated weights (geometric mean)**

Table S7.2. Aggregated criterion weights under the full panel (26 experts) and the consistent subset (15 experts).

| **Criterion** | **w (26)** | **w (15)** | **Share (26)** |
| --- | --- | --- | --- |
| Content quality | 0.527 | 0.492 | 52.69% |
| Instructional design | 0.251 | 0.316 | 25.10% |
| Evaluation feedback | 0.120 | 0.078 | 11.98% |
| Platform function | 0.102 | 0.113 | 10.23% |

Note. Weights were obtained by aggregating individual pairwise comparison matrices as the geometric mean of their log-transformed entries, then applying the row geometric-mean (root-mean) method. Share = wi/Σw × 100, calculated on the 26-expert panel. The top two criteria (content quality and instructional design) retained their rank order in both panels; the rank order of evaluation feedback and platform function reversed after excluding inconsistent experts.

# **7.3 Bootstrap 95% confidence intervals**

Table S7.3. Bootstrap 95% confidence intervals for the aggregated criterion weights.

| **Set** | **Criterion** | **Estimate** | **CI low** | **CI high** | **Width** |
| --- | --- | --- | --- | --- | --- |
| 26 | Content quality | 0.527 | 0.493 | 0.557 | 0.063 |
| 26 | Instructional design | 0.251 | 0.228 | 0.279 | 0.050 |
| 26 | Evaluation feedback | 0.120 | 0.104 | 0.136 | 0.033 |
| 26 | Platform function | 0.102 | 0.079 | 0.130 | 0.050 |
| 15 | Content quality | 0.492 | 0.449 | 0.535 | 0.086 |
| 15 | Instructional design | 0.316 | 0.272 | 0.360 | 0.088 |
| 15 | Evaluation feedback | 0.078 | 0.057 | 0.101 | 0.043 |
| 15 | Platform function | 0.113 | 0.075 | 0.151 | 0.076 |

Note. Nonparametric bootstrap with B = 1,000 resamples drawn with replacement from the experts. Intervals are percentile intervals (2.5th–97.5th). Width = upper limit − lower limit. All intervals exclude zero, indicating that every criterion retains a non-negligible weight. Under the 26-expert panel, content quality showed the widest interval (width = 0.063); under the 15-expert panel, instructional design showed the widest interval (width = 0.088).

# **7.4 Weight deviation after exclusion**

Table S7.4. Sensitivity of criterion weights to the exclusion of inconsistent experts.

| **Criterion** | **w (26)** | **w (15)** | **\|Δw\|** | **Rel. %** |
| --- | --- | --- | --- | --- |
| Content quality | 0.527 | 0.492 | 0.035 | 6.64 |
| Instructional design | 0.251 | 0.316 | 0.065 | 25.90 |
| Evaluation feedback | 0.120 | 0.078 | 0.042 | 35.00 |
| Platform function | 0.102 | 0.113 | 0.011 | 10.78 |

Note. |Δw| = |w(15 experts) − w(26 experts)|; relative deviation = |Δw| / w(26 experts) × 100. The largest absolute change was observed for instructional design (|Δw| = 0.065), whereas the largest relative change was observed for evaluation feedback (35.00%). Platform function showed the smallest absolute change (0.011; 10.78% relative). Content quality remained the highest-weighted criterion in both sets. However, the rank order of evaluation feedback and platform function reversed after excluding inconsistent experts. Overall, the top two criteria were stable, but the lower-weighted criteria were more sensitive in relative terms.

# **7.5 Demographic selective-bias test**

Table S7.5a. Cross-tabulation of experts' professional field by consistency status.

|  | **Medical Education Research** | **Educational Administration** | **Clinical Medicine Expert** | **Teaching Expert** | **Total** |
| --- | --- | --- | --- | --- | --- |
| Pass | 5 | 5 | 2 | 3 | 15 |
| Exclude | 2 | 0 | 5 | 4 | 11 |
| Total | 7 | 5 | 7 | 7 | 26 |

Note. Pearson χ² test, df = 3; χ² = 7.271, asymptotic P = 0.064. Because several cells have expected counts below 5 (minimum expected count = 2.12), a Monte Carlo permutation test (20,000 permutations) was also performed, giving P = 0.078. Both tests indicate no association between professional field and consistency status (no selective bias).

Table S7.5b. Years of professional experience by consistency status.

| **Group** | **n** | **Mean ± SD (yrs)** |
| --- | --- | --- |
| Pass | 15 | 27.20 ± 12.19 |
| Exclude | 11 | 19.18 ± 10.92 |

Note. Values are mean ± SD (years). Groups were compared with a two-sided Mann–Whitney U test: U = 51.5, z = 1.593, normal-approximation P = 0.111; permutation P = 0.113 (20,000 permutations, seed 20260911). The rank-based test is reported because the samples are small and unbalanced. No significant difference in experience was observed between experts who passed and those excluded.
