# Supplementary material for: Development and validation of a digital learning resource quality evaluation framework: a mixed-methods study in health professions education
Source: Front Public Health. 2026 Oct 14;14:1958724. doi: 10.3389/fpubh.2026.1958724 (PMC13715655; doi:10.3389/fpubh.2026.1958724)
Supplement: Supplementary file 13 [file Table_8.DOCX]

**Supplementary material 8:**

**8.1 Delphi Expert Consultation Results**

Table S8.1 First-round Consultation Results.

| Item | Importance Score (M±SD) | Coefficient of Variation | Full-score Percentage (%) | Median (Mdn) | IQR (Q3−Q1) | % Agreement (≥4) |
| --- | --- | --- | --- | --- | --- | --- |
| 1.1.1 Advancement | 4.62 ± 0.64 | 0.14 | 69.23 | 5.00 | 1.00 | 92.31 |
| 1.1.2 Student-centeredness | 4.85 ± 0.37 | 0.08 | 84.62 | 5.00 | 0.00 | 100.00 |
| 1.2.1 Medical Humanities and Ethics | 4.69 ± 0.55 | 0.12 | 73.08 | 5.00 | 0.75 | 96.15 |
| 1.2.2 Responsiveness to Societal Needs | 4.46 ± 0.65 | 0.14 | 53.85 | 5.00 | 1.00 | 92.31 |
| 1.3.1 Scientificity | 4.85 ± 0.37 | 0.08 | 84.62 | 5.00 | 0.00 | 100.00 |
| 1.3.2 Frontier | 4.58 ± 0.70 | 0.15 | 65.38 | 5.00 | 1.00 | 96.15 |
| 1.3.3 High-order | 4.65 ± 0.56 | 0.12 | 69.23 | 5.00 | 1.00 | 96.15 |
| 2.1.1 Methodological Advancement | 4.54 ± 0.65 | 0.14 | 61.54 | 5.00 | 1.00 | 92.31 |
| 2.1.2 Alignment with Curriculum | 4.81 ± 0.40 | 0.08 | 80.77 | 5.00 | 0.00 | 100.00 |
| 2.2.1 Resource Diversity | 4.46 ± 0.90 | 0.2 | 65.38 | 5.00 | 1.00 | 88.46 |
| 2.2.2 Resource Adaptability | 4.54 ± 0.58 | 0.13 | 57.69 | 5.00 | 1.00 | 96.15 |
| 2.2.3 Learner Control | 4.38 ± 0.75 | 0.17 | 53.85 | 5.00 | 1.00 | 84.62 |
| 2.2.4 Measurability of Learning Outcomes | 4.35 ± 0.89 | 0.21 | 53.85 | 5.00 | 1.00 | 88.46 |
| 3.1.1 Accessibility | 3.86 ± 0.99 | 0.26 | 34.62 | 4.00 | 2.00 | 61.54 |
| 3.1.2 Level of Interactive Engagement | 3.92 ± 0.98 | 0.25 | 30.77 | 4.00 | 1.75 | 73.08 |
| 3.1.3 Data Visualization | 4.42 ± 0.58 | 0.13 | 46.15 | 4.00 | 1.00 | 96.15 |
| 3.2.1 Collaborative Learning Function | 3.77 ± 0.95 | 0.25 | 23.08 | 4.00 | 1.00 | 65.38 |
| 4.1.1 Interface Aesthetics | 3.77 ± 0.99 | 0.26 | 23.08 | 4.00 | 1.00 | 69.23 |
| 4.1.2 Information Visualization | 4.23 ± 0.65 | 0.15 | 34.62 | 4.00 | 1.00 | 88.46 |
| 5.1.1 Update Frequency | 4.38 ± 0.80 | 0.18 | 53.85 | 5.00 | 1.00 | 88.46 |
| 5.2.1 Timeliness of Teacher Response | 4.04 ± 1.00 | 0.25 | 42.31 | 4.00 | 2.00 | 69.23 |

Notes on reported metrics: Median (Mdn) = median of expert importance ratings; IQR (Q3-Q1) = interquartile range, indicating the spread of the central 50% of ratings; % Agreement (≥4) = proportion of experts rating the indicator as 4 or 5 on the 5-point scale. This is distinct from Full-score Percentage, which counts only the proportion of experts giving the maximum score (5/5). Together with M, SD, and CV, these additional metrics provide a more complete picture of inter-rater consensus.

Table S8.2 Second-round Consultation Results.

| Item | Importance Score (M±SD) | Coefficient of Variation | Full-score Percentage (%) | Median (Mdn) | IQR (Q3−Q1) | % Agreement (≥4) |
| --- | --- | --- | --- | --- | --- | --- |
| 1.1.1 Advancement | 4.88 ± 0.33 | 0.07 | 88.46 | 5.00 | 0.00 | 100.00 |
| 1.1.2 Student-centeredness | 4.85 ± 0.37 | 0.08 | 84.62 | 5.00 | 0.00 | 100.00 |
| 1.2.1 Medical Humanities and Ethics | 4.77 ± 0.51 | 0.11 | 80.77 | 5.00 | 0.00 | 96.15 |
| 1.2.2 Social Responsiveness & Primary Healthcare Adaptation | 4.58 ± 0.64 | 0.14 | 65.38 | 5.00 | 1.00 | 92.31 |
| 1.3.1 Scientificity | 4.69 ± 0.47 | 0.10 | 69.23 | 5.00 | 1.00 | 100.00 |
| 1.3.2 Frontier | 4.58 ± 0.50 | 0.11 | 57.69 | 5.00 | 1.00 | 100.00 |
| 2.1.1 Methodological Adaptability | 4.69 ± 0.68 | 0.14 | 76.92 | 5.00 | 0.00 | 96.15 |
| 2.2.1 Resource Diversity | 4.81 ± 0.40 | 0.08 | 80.77 | 5.00 | 0.00 | 100.00 |
| 2.3.1 Personalized Learning | 4.58 ± 0.58 | 0.13 | 61.54 | 5.00 | 1.00 | 96.15 |
| 3.1.1 Multiple Evaluations | 4.50 ± 0.65 | 0.14 | 57.69 | 5.00 | 1.00 | 92.31 |
| 3.2.1 Update Timeliness and Adaptability | 4.42 ± 0.58 | 0.13 | 46.15 | 4.00 | 1.00 | 96.15 |
| 4.1.1 Interface Friendliness | 4.19 ± 0.80 | 0.19 | 42.31 | 4.00 | 1.00 | 76.92 |
| 4.1.2 Data Visualization | 4.54 ± 0.51 | 0.11 | 53.85 | 5.00 | 1.00 | 100.00 |
| 4.2.1 Support for Interaction | 4.19 ± 0.69 | 0.17 | 34.62 | 4.00 | 1.00 | 84.62 |

Notes on reported metrics: Median (Mdn) = median of expert importance ratings; IQR (Q3-Q1) = interquartile range, indicating the spread of the central 50% of ratings; % Agreement (≥4) = proportion of experts rating the indicator as 4 or 5 on the 5-point scale. This is distinct from Full-score Percentage, which counts only the proportion of experts giving the maximum score (5/5). Together with M, SD, and CV, these additional metrics provide a more complete picture of inter-rater consensus.

Table S8.3 Item-level content validity index (I-CVI) for the 14 tertiary indicators (Round 2, n = 26).

| No. | Final tertiary indicator | Corresponding item in Table S8.2(Round 2) | A/N | I-CVI | k* | Exact binomial P | Decision |
| --- | --- | --- | --- | --- | --- | --- | --- |
| 1.1.1 | Advancedness | 1.1.1 Advancement | 26/26 | 1.000 | 1.000 | 1.5×10⁻⁸ | Retained |
| 1.1.2 | Student-Centeredness | 1.1.2 Student-centeredness | 26/26 | 1.000 | 1.000 | 1.5×10⁻⁸ | Retained |
| 1.2.1 | Scientific Accuracy | 1.3.1 Scientificity | 26/26 | 1.000 | 1.000 | 1.5×10⁻⁸ | Retained |
| 1.2.2 | Updated Content & Frontier Knowledge | 1.3.2 Frontier | 26/26 | 1.000 | 1.000 | 1.5×10⁻⁸ | Retained |
| 1.3.1 | Professional Ethics & Values Education | 1.2.1 Medical Humanities and Ethics | 25/26 | 0.962 | 0.962 | 4.0×10⁻⁷ | Retained |
| 1.3.2 | Social Responsiveness | 1.2.2 Social Responsiveness & Primary  Healthcare Adaptation | 24/26 | 0.923 | 0.923 | 5.2×10⁻⁶ | Retained |
| 2.1.1 | Methodological Appropriateness | 2.1.1 Methodological Adaptability | 25/26 | 0.962 | 0.962 | 4.0×10⁻⁷ | Retained |
| 2.2.1 | Resource Formats | 2.2.1 Resource Diversity | 26/26 | 1.000 | 1.000 | 1.5×10⁻⁸ | Retained |
| 2.3.1 | Personalized Learning | 2.3.1 Personalized Learning | 25/26 | 0.962 | 0.962 | 4.0×10⁻⁷ | Retained |
| 2.3.2 | Interaction Design | 4.2.1 Support for Interaction | 22/26 | 0.846 | 0.846 | 2.7×10⁻⁴ | Retained |
| 3.1.1 | Multiple Evaluations | 3.1.1 Multiple Evaluations | 24/26 | 0.923 | 0.923 | 5.2×10⁻⁶ | Retained |
| 3.2.1 | Timeliness & Update Adaptability | 3.2.1 Update Timeliness and Adaptability | 25/26 | 0.962 | 0.962 | 4.0×10⁻⁷ | Retained |
| 4.1.1 | Compatibility & Security | 4.1.1 Interface Friendliness | 20/26 | 0.769 | 0.768 | 4.7×10⁻³ | Retained  (exception rule) |
| 4.1.2 | Data Visualization | 4.1.2 Data Visualization | 26/26 | 1.000 | 1.000 | 1.5×10⁻⁸ | Retained |

I-CVI = importance-based content validity index (proportion of experts rating ≥ 4 on the 5-point importance scale); k* = modified kappa (Polit & Beck, 2006); 0.78 is retained as a conservative screening benchmark. Source of raw counts: Supplementary Material 8, Table S8.2, column "% Agreement (≥4).

# 8.2 Four-Quadrant Threshold Sensitivity Analysis

This section reports the sensitivity analysis of indicator classification in the four-quadrant prioritization matrix. The measurability threshold was varied from 3.8 to 4.2 (in increments of 0.1) while the integrated weight threshold was held constant at 0.06, to assess the stability of quadrant assignments near the pre-specified cut-off of 4.0.

Table S8.4 Number of indicators per quadrant across measurability thresholds

| Measurability Threshold | Quadrant I | Quadrant II | Quadrant III | Quadrant IV | No. of Indicators Changing Quadrant |
| --- | --- | --- | --- | --- | --- |
| 3.8 | 9 | 0 | 0 | 5 | 2 |
| 3.9 | 9 | 0 | 0 | 5 | 2 |
| 4.0 (baseline) | 7 | 2 | 0 | 5 | 0 |
| 4.1 | 6 | 3 | 3 | 2 | 4 |
| 4.2 | 6 | 3 | 3 | 2 | 4 |

Table S8.5 Indicators changing quadrant across measurability thresholds

| **Threshold Range** | **Indicators Changed** | **Direction of Movement** |
| --- | --- | --- |
| 3.8–3.9 | Data Visualization, Compatibility & Security | II → I |
| 4.1–4.2 | Resource Formats | I → II |
| 4.1–4.2 | Social Responsiveness, Personalized Learning, Timeliness & Update Adaptability | IV → III |

## Summary

Across the full range of measurability thresholds (3.8–4.2), the overall quadrant structure remained reasonably stable. At thresholds of 3.8–3.9, the two Quadrant II indicators (Data Visualization, Compatibility & Security) moved to Quadrant I as their measurability scores exceeded the lowered threshold. At thresholds of 4.1–4.2, four indicators shifted quadrants: Resource Formats moved from Quadrant I to II, while Social Responsiveness, Personalized Learning, and Timeliness & Update Adaptability moved from Quadrant IV to III. Across the full range, the high-priority Quadrant I set retained at least 6 of its 7 baseline indicators, and no indicator moved more than one quadrant away from its baseline classification.

**8.3 Entropy Weight Method (EWM) input matrix — descriptives**

The data-derived weighting derives its weights from the same Round-2 expert rating matrix used for the Delphi consensus criteria. To help readers assess the underlying dispersion structure that drives the EWM weights, the following descriptive statistics are reported for the matrix (26 raters × 14 indicators, n = 364 cells, all entries on a 5-point Likert scale).

Table S8.6 Descriptive statistics of the Round 2 expert rating matrix used for EWM computation

| **Statistic** | **Value** |
| --- | --- |
| Mean of all cell entries (M̄) | 4.59— overall mean importance score across all raters × indicators |
| Standard deviation of all cell entries (SD) | 0.59— dispersion that drives the EWM weights |
| Minimum / Maximum cell values | 2/5— all entries are on a 1–5 Likert scale |
| Number (and %) of cells with value = 5 (top score) | 234 (64.3%)— indicates strong endorsement instances |
| Number (and %) of cells with value ≤ 2 (low score) | 1 (0.27%)— indicates weak endorsement instances |
| Cronbach's α of the 26 raters' rating profile (Round 2) | 0.78— within-rater reliability (rater-as-item) |
