# Supplementary material for: Development and validation of a digital learning resource quality evaluation framework: a mixed-methods study in health professions education
Source: Front Public Health. 2026 Oct 14;14:1958724. doi: 10.3389/fpubh.2026.1958724 (PMC13715655; doi:10.3389/fpubh.2026.1958724)
Supplement: Supplementary file 14 [file Table_9.DOCX]

# Supplementary Material 9: Full Results of Sensitivity Analysis for the Integrated Weighting Model

## 9.1 Methods

The integrated weight of each indicator was defined as W(β) = β × W(AHP) + (1 − β) × W(EWM), where W(AHP) denotes the AHP weight, W(EWM) the data-derived entropy weight computed from the Round 2 expert rating matrix, and β = 0.5 in the main analysis (equal contribution of both components). To assess the sensitivity of the framework to the choice of β, all weights were recomputed at β = 0.3, 0.4, 0.5, 0.6, and 0.7. Ranking stability was evaluated against the baseline (β = 0.5) using Spearman rank correlation coefficients and the number of indicators changing rank. The analysis was performed at the primary(n = 4) and tertiary(n = 14) levels; the secondary level serves as an intermediate classification layer and is not weighted or reported separately. Global tertiary AHP weights were derived from the hierarchical structure (primary × secondary × within-secondary local weights), consistent with Table 4 of the main text.

## 9.2 Primary-dimension level (n = 4)

Table S9.1 Integrated weights of the primary dimensions across β = 0.3–0.7.

| **Primary Dimension** | **AHP Weight** | **Data-derived (EWM) Weight** | **W (β = 0.3)** | **W (β = 0.4)** | **W (β = 0.5)** | **W (β = 0.6)** | **W (β = 0.7)** |
| --- | --- | --- | --- | --- | --- | --- | --- |
| Content Quality | 0.492 | 0.445 | 0.459 (1) | 0.464 (1) | 0.469 (1) | 0.473 (1) | 0.478 (1) |
| Instructional Design | 0.316 | 0.184 | 0.224 (3) | 0.237 (2) | 0.250 (2) | 0.263 (2) | 0.276 (2) |
| Evaluation & Feedback | 0.078 | 0.072 | 0.074 (4) | 0.074 (4) | 0.075 (4) | 0.076 (4) | 0.076 (4) |
| Platform Functionality | 0.113 | 0.299 | 0.243 (2) | 0.225 (3) | 0.206 (3) | 0.187 (3) | 0.169 (3) |

Note: Values in parentheses denote the rank at each β; the baseline (β = 0.5) is shown in bold in the main text.

Primary-dimension rankings were identical to the baseline across β = 0.4–0.7 (Spearman ρ = 1.00). At β = 0.3, a single adjacent rank exchange occurred between Instructional Design and Platform Functionality (ranks 2 and 3; ρ = 0.80). Because only four primary dimensions are compared, the exact two-tailed p-value for ρ = 1.00 at n = 4 is 0.083 (the minimum achievable); rank correlations at this level are therefore interpreted descriptively rather than statistically.

## 9.3 Tertiary-indicator level (n = 14)

Table S9.2 Integrated weights of the 14 tertiary indicators across β = 0.3–0.7.

| Tertiary Indicator | AHP Weight (global) | Data-derived (EWM) Weight | W (β = 0.3) | W (β = 0.4) | W (β = 0.5) | W (β = 0.6) | W (β = 0.7) |
| --- | --- | --- | --- | --- | --- | --- | --- |
| 1.1.1 Advancedness | 0.078 | 0.039 | 0.051 (9) | 0.055 (10) | 0.059 (10) | 0.062 (9) | 0.066 (8) |
| 1.1.2 Student-Centeredness | 0.136 | 0.053 | 0.078 (5) | 0.086 (4) | 0.095 (3) | 0.103 (2) | 0.111 (1) |
| 1.2.1 Scientific Accuracy | 0.066 | 0.118 | 0.102 (3) | 0.097 (3) | 0.092 (4) | 0.087 (5) | 0.082 (5) |
| 1.2.2 Updated Content & Frontier Knowledge | 0.031 | 0.176 | 0.133 (2) | 0.118 (2) | 0.104 (2) | 0.089 (4) | 0.075 (7) |
| 1.3.1 Professional Ethics & Values Education | 0.116 | 0.02 | 0.049 (10) | 0.058 (9) | 0.068 (8) | 0.078 (7) | 0.087 (4) |
| 1.3.2 Social Responsiveness | 0.065 | 0.038 | 0.046 (11) | 0.049 (11) | 0.052 (11) | 0.054 (11) | 0.057 (10) |
| 2.1.1 Methodological Appropriateness | 0.145 | 0.016 | 0.055 (8) | 0.068 (7) | 0.081 (5) | 0.093 (3) | 0.106 (3) |
| 2.2.1 Resource Formats | 0.051 | 0.068 | 0.063 (7) | 0.061 (8) | 0.060 (9) | 0.058 (10) | 0.056 (11) |
| 2.3.1 Personalized Learning | 0.037 | 0.027 | 0.030 (14) | 0.031 (14) | 0.032 (13.5) | 0.033 (14) | 0.034 (13) |
| 2.3.2 Interaction Design | 0.083 | 0.073 | 0.076 (6) | 0.077 (5) | 0.078 (6) | 0.079 (6) | 0.080 (6) |
| 3.1.1 Multiple Evaluations | 0.045 | 0.041 | 0.042 (12) | 0.043 (12) | 0.043 (12) | 0.043 (12) | 0.044 (12) |
| 3.2.1 Timeliness & Update Adaptability | 0.033 | 0.031 | 0.032 (13) | 0.032 (13) | 0.032 (13.5) | 0.032 (13) | 0.032 (14) |
| 4.1.1 Compatibility & Security | 0.04 | 0.101 | 0.083 (4) | 0.077 (6) | 0.071 (7) | 0.064 (8) | 0.058 (9) |
| 4.1.2 Data Visualization | 0.073 | 0.198 | 0.161 (1) | 0.148 (1) | 0.136 (1) | 0.123 (1) | 0.111 (2) |

Note: Values in parentheses denote the rank at each β. Personalized Learning and Timeliness & Update Adaptability have identical integrated weights at β = 0.5 (W = 0.0320) and were therefore both assigned the average rank of 13.5; rank-change counts in Table S9.3 are calculated relative to these average baseline ranks.

Tertiary-indicator rankings were highly correlated with the baseline across the entire range: Spearman ρ = 0.928 at β = 0.3 and 0.858 at β = 0.7 (both P < 0.001), with 9, 9, 10, and 12 indicators changing rank at β = 0.3, 0.4, 0.6, and 0.7, respectively. Most changes involved movements of one to two positions among indicators separated by less than 0.01 in weight, although two indicators moved further at β = 0.7. The largest movement involved Updated Content & Frontier Knowledge (1.2.2), which combines the second-highest data-derived weight (0.176) with a low global AHP weight (0.031) and therefore shifted from rank 2 at baseline to rank 7 at β = 0.7; Professional Ethics & Values Education (1.3.1) moved in the opposite direction (rank 8 → 4). Data Visualization (4.1.2), which combines the highest data-derived weight (0.198) with a moderate global AHP weight (0.073), retained rank 1 from β = 0.3 through β = 0.6 and moved to rank 2 at β = 0.7, exchanging with Student-Centeredness (1.1.2). Indicators crossing the 0.06 reference value used in the quadrant analysis (Advancedness, Methodological Appropriateness, Professional Ethics & Values Education, Resource Formats, Compatibility & Security) should be interpreted as specification-sensitive.

## 9.4 Summary of ranking stability

Table S9.3 Rank stability across β settings: Spearman rank correlations and rank-change counts versus the β = 0.5 baseline.

| **Level** | **β** | **Spearman ρ vs. baseline** | **Indicators changing rank** | **p-value** |
| --- | --- | --- | --- | --- |
| Primary (n = 4) | 0.3 | 0.80 | 2 (ID ↔ PF, adjacent) | 0.333 (exact) |
| Primary (n = 4) | 0.4 | 1.00 | 0 | 0.083 (exact) |
| Primary (n = 4) | 0.5 | — (baseline) | 0 | — |
| Primary (n = 4) | 0.6 | 1.00 | 0 | 0.083 (exact) |
| Primary (n = 4) | 0.7 | 1.00 | 0 | 0.083 (exact) |
| Tertiary (n = 14) | 0.3 | 0.928 | 9 | < 0.001 |
| Tertiary (n = 14) | 0.4 | 0.977 | 9 | < 0.001 |
| Tertiary (n = 14) | 0.5 | — (baseline) | 0 | — |
| Tertiary (n = 14) | 0.6 | 0.968 | 10 | < 0.001 |
| Tertiary (n = 14) | 0.7 | 0.858 | 12 | < 0.001 |

Note. With n = 4, the smallest achievable exact two-tailed p-value is 0.083; primary-level correlations are therefore reported for descriptive purposes. Rank-change counts at the tertiary level include the reordering of the two indicators tied at baseline (Personalized Learning and Timeliness & Update Adaptability).

## 9.5 Interpretation

Across β = 0.3–0.7, the integrated weighting results and the resulting prioritization of indicators were robust: almost all rank changes were single adjacent exchanges among near-tied indicators, although exact ordinal positions were not invariant to β (9–12 indicators changed rank), and the high-priority set identified by the four-quadrant analysis was substantially preserved (at least 6 of the 7 baseline Quadrant I indicators were retained across the tested measurability thresholds; Supplementary Material 8, Section 8.2), although five indicators with baseline weights adjacent to the 0.06 reference value crossed that threshold at one or more β settings. The equal-contribution default $\text{β=0.5}$ therefore remains a transparent and neutral specification and does not materially drive the conclusions of this study. Indicators whose weights lie close to the 0.06 reference value used in the quadrant analysis (particularly Advancedness and Resource Formats at baseline) should, however, be interpreted as borderline or potentially sensitive to the weighting specification, especially those with large AHP–EWM differences, regardless of β.
